# Comparative cytoarchitectural and proteomic analysis of the primary motor cortex in pigs and wild boars reveals domestication associated changes

**DOI:** 10.7717/peerj.21640

**Published:** 2026-08-18

**Authors:** Giulia Lazzarini, Maurizio Ronci, Lorenzo Zallocco, Federica Di Cintio, Daniela Beghelli, Vincenzo Miragliotta, Chiara Magliaro, Carlo Cantile, Laura Giusti, Andrea Pirone

**Affiliations:** 1 Department of Veterinary Sciences, University of Pisa, Pisa, Italy; 2 Department of Medical, Oral and Biotechnological Sciences, University of Chieti, Chieti, Italy; 3 COIIM—Interuniversitary Consortium for Engineering and Medicine, Campobasso, Italy; 4 Department of Translational Research and New Technologies in Medicine and Surgery, University of Pisa, Pisa, Italy; 5 School of Biosciences and Veterinary Medicine, University of Camerino, Camerino, Italy; 6 Department of Information Engineering, University of Pisa, Pisa, Italy; 7 Research Center “E. Piaggio”, University of Pisa, Pisa, Italy; 8 Department of Pharmacy and Biotechnologies, University of Bologna, Bologna, Italy

**Keywords:** Domestication, Primary motor cortex, Parvalbumin, Pigs, Proteomic, Wild boars

## Abstract

**Background:**

Domestication appears to modify the morphology and physiology of the central nervous system. Owing to the limited availability of proteomic and cytoarchitectural data for comparisons between wild and domesticated species, we conducted a comparative analysis of the cytoarchitecture and protein profile of the primary motor cortex (M1), the key region controlling motor activity, in pigs and wild boars to assess the effects of domestication.

**Methods:**

M1 samples used in this study were obtained from brains previously analyzed in an earlier investigation. From six pig and wild boar brains fixed in paraformaldehyde, the right and left M1 regions were isolated. Paraffin sections, 5 µm thick, were prepared for histological and immunohistochemical analyses, and 10 µm sections were used for proteomic analysis. In M1, cortical thickness, cell density, and the density of parvalbumin-positive neurons were quantified, while proteomic analysis was performed to characterize the M1 protein profile.

**Results:**

Our results revealed a lower density of parvalbumin-expressing interneurons compared with wild boars. Moreover, proteomic analyses showed an overexpression in wild boars of proteins involved in oxidative stress protection and synaptic plasticity.

**Conclusions:**

These findings indicate that domestication may have influenced the cytoarchitecture of the pig M1. The reduced number of parvalbumin-expressing interneurons may reflect a modification in neuronal network properties in pigs compared with wild boars. In contrast, the proteomic profile of wild boars revealed an enrichment of proteins associated with oxidative stress protection and synaptic plasticity, supporting structural distinctions in the M1. Collectively, these results suggest that the wild boar may exhibit more finely tuned regulation of motor control, supported by enhanced mechanisms to sustain neuronal activity and viability.

## Introduction

The motor cortex, first mapped in dogs by Fritsch and Hitzig in 1870 (Fritsch & Hitzig, 2009), is essential for controlling motor activity, contributing to both movement generation and inhibition. Owing to this fundamental role, its organization has been largely conserved across mammals (Ebbesen & Brecht, 2017). The cytoarchitecture of the motor cortex is typically characterized by five cortical layers, with layer 4 nearly absent and a prominent layer 5 containing large pyramidal neurons, known as Betz cells in primates (Bakken et al., 2021). In other mammals, such as sheep (Ebinger, 1975), cetaceans (Kesarev, Malofeeva & Trykova, 1977; Hof & Gucht, 2007), and giraffes (Badlangana et al., 2007), large pyramidal neurons are also present but are smaller than Betz cells.

The mammalian cortex mainly comprises two neuronal populations: glutamatergic excitatory neurons and GABAergic inhibitory interneurons. The activity of GABAergic interneurons is critical for cortical information processing. Within the primary motor cortex, parvalbumin (PV)-expressing GABAergic interneurons play a major role in modulating the excitatory output of pyramidal neurons, thereby refining motor signaling (Tremblay, Lee & Rudy, 2016; Estebanez et al., 2017).

Together with the cerebellum, the primary motor cortex (M1) represents one of the main neural structures governing motor output, essential for adaptive behavior and survival (Apps & Garwicz, 2005; Ebbesen & Brecht, 2017). In this context, we recently investigated the development of the M1 in pigs (Desantis et al., 2021) and compared the cerebellum of wild boars and pigs to explore possible effects of domestication (Pirone et al., 2022). This question is particularly relevant, given that domestic pigs (*Sus scrofa* domesticus) originated from Eurasian wild boars (*Sus scrofa*) (Giuffra et al., 2000; Larson et al., 2005).

Domestication can be broadly defined as a specific evolutionary process involving prolonged interaction and selective pressure between humans and another species, often viewed as a form of interspecific cooperative breeding (Mueller & Willman, 2024). Domestication is thought to modify the morphology and physiology of the central nervous system (CNS). Macroscopic data generally indicate that domesticated species possess smaller brains than their wild counterparts (Castiglione et al., 2020; Balcarcel et al., 2022). In pigs, brain volume is on average 33.6% smaller than in wild boars (Kruska, 2005), although more recent endocast-based measurements suggest a reduction of about 18% (Cucchi et al., 2024). Nevertheless, quantitative information on molecular, microstructural, and neurochemical differences between wild and domesticated animals remains scarce.

Here, we compared the cytoarchitecture and proteomic profile of the M1 in pigs and wild boars to evaluate the potential influence of domestication on this cortical region.

## Materials and Methods

### Animals and tissue sampling

The M1 samples used in this study were obtained from brains previously analyzed in an earlier investigation (Pirone et al., 2022); no additional animals were sacrificed specifically for the present work. Brains from six adult pigs (*Sus scrofa domesticus*, 170–175 kg, >10 months) and six adult wild boars (*Sus scrofa*, 63.33 ± 12.11 kg, >12 months) were examined. Sex information was unavailable for pigs, whereas the wild boar group included three males and three females.

Age estimation for wild boars followed the method described by Drimaj et al. (2020). Pigs were slaughtered for commercial purposes at a local abattoir (Desideri Luciano s.r.l., *Via* Abruzzi 2, 56025 Pontedera, Tuscany, Italy) in accordance with European Regulation (EC 1099/2009) on animal welfare during commercial slaughter, under the supervision of official public health veterinarians. All animals were in good body condition and showed no pathological alterations at ante-mortem or post-mortem inspection. Wild boar specimens were obtained post-mortem from routine hunting activities conducted in accordance with regional law no. 3-1994 and national law no. 157-1992.

**Figure 1:**
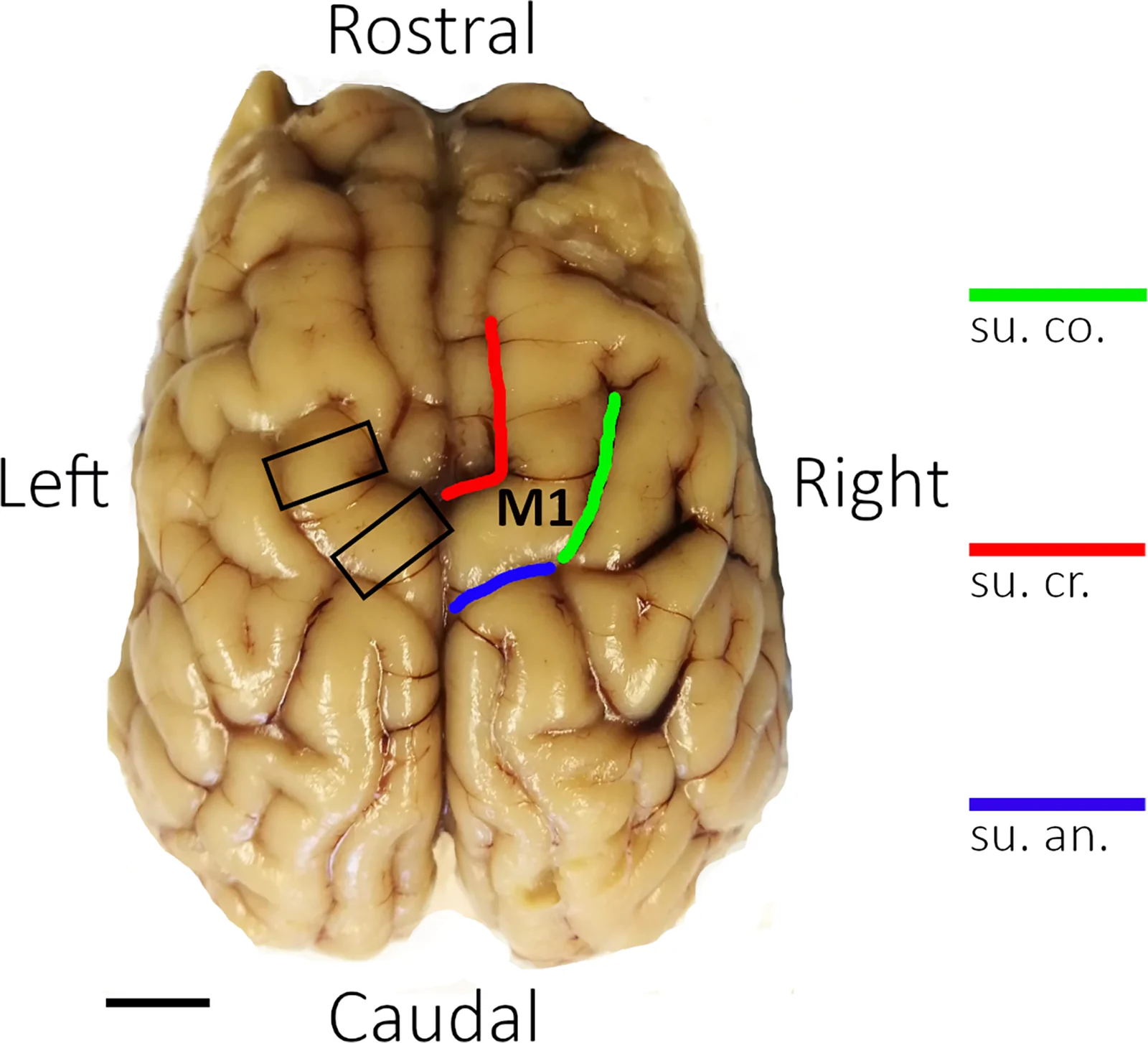
Anatomical localization of the primary motor cortex (M1). Dorsal view of a pig brain showing the location of the M1 identified based on the position of the *sulcus cruciatus* (*su. cr.), sulcus ansatus* (*su. an*.), and *sulcus coronalis* (*su. co*.). Black rectangles on the left M1 indicate approximately the levels of the two sampled tissue blocks. Scale bar = 1 cm.

Brains were extracted and immersion-fixed for 3 months in 4% paraformaldehyde in 0.1 M phosphate-buffered saline (PBS, pH 7.4) at room temperature, with the fixative renewed after 45 days. The brains were then weighed, and the corresponding values were reported in Table S1. The M1 (left and right hemispheres) was identified according to the position of the *sulcus cruciatus*, *sulcus coronalis*, and *sulcus ansatus* (Fig. 1) (Palmieri et al., 1986; Ernst et al., 2018; del Cerro, Rodríguez-De-Lope & Collazos-Castro, 2021). Two tissue blocks, approximately 1 cm apart, were collected from each hemisphere and processed for paraffin embedding. From each block, 5 µm-thick paraffin sections were cut for histological and immunohistochemical analyses, while 10 µm-thick sections were prepared for proteomic analysis.

### Histological and immunohistochemical staining

To examine the general neuronal architecture of the motor cortex, sections were stained using the Nissl method with cresyl violet. After deparaffinization in xylene and rehydration through graded ethanol, sections were stained with 0.1% cresyl violet (C1791; Sigma-Aldrich, St. Louis, MO, USA) for 5 min, rinsed in distilled water, differentiated in 95% ethanol for 2–10 min, dehydrated in 100% ethanol, cleared in xylene, and mounted with permanent mounting medium (06522; Sigma-Aldrich, St. Louis, MO, USA).

Immunoperoxidase staining was performed on serial 5 µm sections using a mouse monoclonal anti-parvalbumin antibody (PV, 1:2,000; details in Table S2). Epitope retrieval was carried out at 120 °C for 5 min in a pressure cooker using Tris/EDTA buffer (pH 9.0). Sections were incubated with 1% H_2_O_2_ in PBS for 10 min to quench endogenous peroxidase, rinsed three times in 0.05% Triton X-100 in PBS (3 × 10 min), and blocked for 1 h with 5% normal horse serum (PK-7200; Vector Labs, Newark, CA, USA) in PBS. Sections were then incubated overnight at 4 °C with the primary antibody in 2% normal horse serum and 0.05% Triton X-100 in PBS. After rinsing in PBS (3 × 10 min), slides were incubated with biotinylated horse anti-mouse IgG (1:300; details in Table S2) followed by the Vectastain Elite ABC-HRP reagent (PK-7200; Vector Labs, Newark, CA, USA). The reaction product was visualized using diaminobenzidine (ImmPACT DAB Substrate Kit, SK-4105; Vector Labs, Newark, CA, USA). Specificity of immunostaining was verified using negative controls in which the primary antibody, secondary antibody, or ABC complex was replaced with PBS or non-immune serum. Under these conditions, no non-specific staining was observed. According to the manufacturer, the anti-PV antibody cross-reacts with porcine parvalbumin.

### Image acquisition and morphometry

Measurements were obtained from four sections per hemisphere (left and right) per animal. The first two sections and the second two were sampled from the caudal and rostral tissue blocks, respectively. Sections within each pair were spaced 200 µm apart, and the distance between pairs was approximately 1 cm. Whole-slide images were acquired using a NanoZoomer Hamamatsu slide scanner at 20× magnification with automatic focusing. Quantitative morphometric parameters extracted from each set of acquired images are reported in Table S3.

### M1 thickness

M1 thickness (M1T) was measured using Aperio ImageScope software (Leica Biosystems Imaging). On each whole-slide image, two reference lines were manually drawn along the pial surface and the gray matter (GM)/white matter (WM) interface. The software then automatically generated 25 perpendicular segments connecting the two lines (Fig. S1). Because the GM/WM boundary was difficult to define at the gyral crown, measurements were restricted to the straight cortical regions.

### Density of PV-immunoreactive neurons

Density of PV-immunoreactive neurons (DPVN) was quantified using ImageScope software. The region of interest (ROI) corresponding to the GM was manually delineated (Fig. S2A), and all labeled cell bodies within the ROI were counted (Fig. S2B). Each detected object was automatically labeled to prevent duplicate counting (Fig. S2C).

### Density of cells (DC)

Due to the high number of Nissl-stained neurons, density of cells (DC) was estimated using Stereo Investigator–Whole Slide Edition (MBF Bioscience), treating the sections as a 2D system. The ROI, extending from the pial surface to the GM/WM interface, was outlined using the contour tool (Fig. S3A). Counting frames (400 × 400 µm) were distributed systematically and randomly across the ROI (Fig. S3B). Each frame included two inclusion (green) and two exclusion (red) borders (Fig. S3C). A cell was counted if its soma lay entirely within the frame or touched an inclusion line. Only neurons with a visible nucleus were included (Fig. S3D).

### Statistical analysis

Graphing and statistical analyses were performed using GraphPad Prism 9.5.1 for Windows (GraphPad Software). Data normality was assessed with the Shapiro–Wilk test. Only measurements from the same animal were treated as paired. To avoid pseudo replication, all morphometric values were averaged per animal and *n* = 6 was used for statistical testing. Depending on distribution and pairing, the following tests were applied, with significance set at *p* < 0.05:

(a) paired t-test for normally distributed paired data;
(b) paired Wilcoxon test for non-normal paired data;
(c) unpaired t-test for normally distributed unpaired data; and
(d) Mann–Whitney test for non-normal unpaired data.

To assess the magnitude of M1T observed differences, effect sizes were calculated using Cohen’s d. For within-animal comparisons between left and right M1T, paired Cohen’s d was derived from the mean of the within-subject differences divided by their standard deviation. For between-species comparisons, Cohen’s d was calculated using the difference between the group means normalized to the pooled standard deviation. Effect sizes were interpreted using standard benchmarks (small, ~0.2; medium, ~0.5; large, ~0.8).

### Proteomic analysis

#### Protein extraction

Protein extraction was performed essentially as described previously (Donadio et al., 2011). Briefly, ten 10 µm-thick sections from each cortical hemisphere (right and left) of pigs (*n* = 3) and wild boars (*n* = 3) were pooled. Sections were deparaffinized with 2–5 changes of xylene (10 min each) and rehydrated through graded ethanol (100% × 2, 85%, 70%; 10 min each). Tissues were then resuspended in extraction buffer (20 mM Tris-HCl, pH 6, containing 2% SDS and 0.2 M glycine) and incubated for 1 h at 4 °C under agitation. Homogenates were sonicated three times for 30 s, heated at 100 °C for 20 min and subsequently at 60 °C for 2 h under agitation.

The extracts were clarified by centrifugation (14,000×g, 20 min, 4 °C). The pellet was resuspended in extraction buffer (pH 8.8), incubated for 3 h at 50 °C, and centrifuged again under the same conditions. Supernatants were combined, and SDS content was reduced using the SDS-Out Precipitation Kit (Thermo Fisher Scientific, Waltham, MA, USA) following the manufacturer’s protocol. The protein content was determined by the RC/DC Protein Assay (Bio-Rad, Hercules, CA, USA) using bovine serum albumin as standard. Samples were stored at –80 °C until analysis.

#### Mass spectrometry

Aliquots containing 50 µg of protein were loaded onto Nanosep 10 kDa cutoff filters (Pall Corporation, MI, USA) and digested as described previously (Pontifex et al., 2024). Samples were washed twice with 200 µL urea buffer (8 M urea, 100 mM Tris, pH 8.5), reduced with 100 µL Dithiothreitol (DTT, 8 mM in urea buffer), and alkylated with 100 µL Iodoacetamide (IAA, 50 mM in urea buffer). Urea was exchanged with 50 mM ammonium bicarbonate before adding trypsin at a 1:50 enzyme-to-protein ratio. Digestion proceeded overnight at 37 °C, after which peptides were collected by centrifugation, acidified with 10% Trifluoroacetic acid (TFA), and stored at –20 °C. Each digested sample was analyzed in technical triplicate by LC-MS/MS using an UltiMate 3000 RSLCnano system (Thermo Fisher Scientific, Waltham, MA, USA) coupled to an Orbitrap Fusion Tribrid mass spectrometer (positive ionization, nanoESI source). Peptides were loaded on a PepMap100 C18 pre-column (5 µm, 100 Å, 300 µm × 5 mm) and separated on an EASY-Spray PepMap RSLC C18 column (2 µm, 100 Å, 75 µm × 15 cm) at 300 nL/min and 40 °C. The chromatographic gradient was: 95% A (0.1% formic acid in water) to 25% B (99.9% acetonitrile, 0.1% formic acid) in 40 min; 25–55% B in 5 min; and 55–90% B in 1 min (total run = 65 min).

Precursor (MS1) scans were recorded in the Orbitrap (resolution 240k @ m/z 200). Data-dependent MS/MS (MS2) was acquired in top-speed mode (3 s cycle), selecting multiply charged (2+–5+) precursors within 375–1,500 m/z. Quadrupole isolation width was 1.6 m/z; dynamic exclusion = 60 s. AGC targets were 4.0 × 10^5^ (MS1) and 1.5 × 10^4^ (MS2) with 50 and 70 ms maximum injection times, respectively. HCD fragmentation used 30% normalized collision energy. Fragment ions were detected in the ion trap (m/z 300–1,200).

#### Bioinformatics

Raw data were processed using PEAKS Studio Xpro (Bioinformatics Solutions Inc., Waterloo, Canada) with the “correct precursor only” option. Spectra were searched against the UniProt SwissProt *Sus scrofa* reference database (23,027 entries) with common contaminants appended. Post-translational modifications (PTMs) were defined as follows: fixed carbamidomethylation (Cys, +57.02 Da); variable oxidation (Met, +15.99 Da), dioxidation (+31.99 Da), deamidation (Asn/Gln, +0.98 Da), methylation (protein N-terminus, +14.02 Da), and formylation (Lys and protein N-terminus, +27.99 Da). Trypsin specificity was applied, allowing ≤2 missed cleavages and ≤3 variable PTMs per peptide. Mass tolerances were set to 10 ppm (precursors) and 0.5 Da (fragments). False discovery rate (FDR) was limited to 5% at the peptide level. Label-free quantification (LFQ) was performed in PEAKS Studio with the following parameters: quantification type = label-free; mass error tolerance = 10 ppm; retention-time shift = auto; FDR = 5%. Differential expression was evaluated by analysis of variance (ANOVA) using thresholds of –10 lgP ≥20, fold change ≥1.2, and ≥1 quantified peptide per protein. Protein abundances were normalized to cytoplasmic actin (Q6QAQ1; ACTB_PIG). Functional enrichment analysis was carried out using STRING v12.0 (June 26, 2023; https://string-db.org/) with the *Sus scrofa* dataset.

## Results

### Brain weight differences between pigs and wild boars

Brain weight differed markedly between groups (Table 1). Pigs exhibited a 26.05% reduction in brain weight relative to wild boars (mean ± s.d.; percentage calculated relative to the wild boar mean). This difference was highly significant, as assessed by a two-tailed Welch’s t-test (*p* < 0.001), indicating a robust group effect independent of variance heterogeneity.

**Table 1:** Summary statistics of brain weight (bw) in pigs (p) and wild boars (wb), reported as mean ± s.d.

Group	*n*	bw (g), mean ± SD	% Difference *vs*. wb	t (Welch)	*p*-value
*p*	6	110.07 ± 3.00	−26.05%		
wb	6	148.83 ± 7.27		−12.07	8.99 × 10^−6^

**Notes:**

Intergroup differences were evaluated using a two-tailed Welch’s t-test.

Brain weight in pigs was significantly lower than in wild boars (−26.05% relative to the wild boar mean; *p* < 0.001).

### Histology and immunohistochemistry

**Figure 2:**
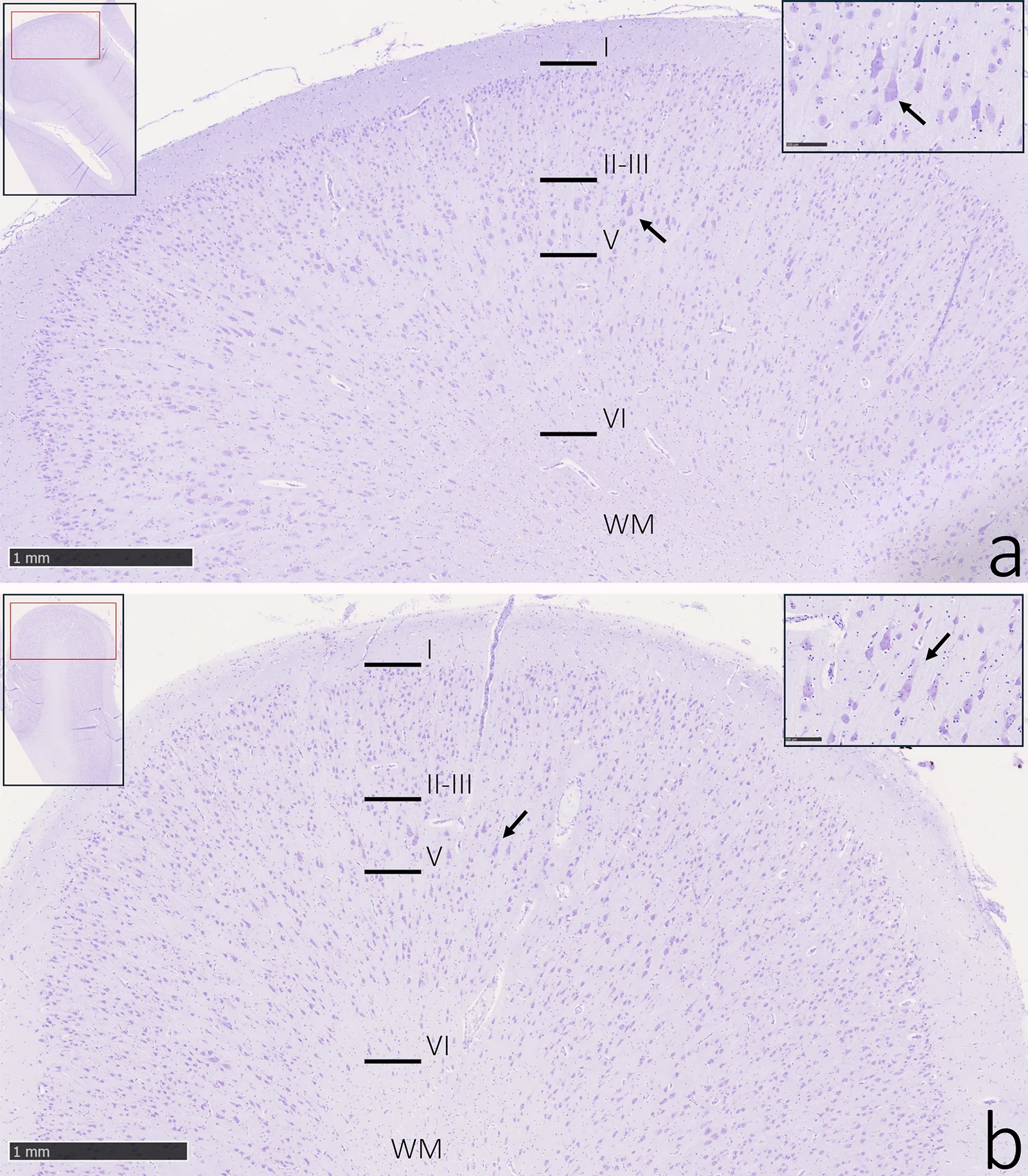
Cytoarchitecture of the primary motor cortex in wild boars and pigs. Nissl-stained sections illustrating the cytoarchitecture of the primary motor cortex in wild boars (A) and pigs (B). Insets in the upper left corners show the M1 gyrus, with red rectangles indicating the region in (A) and (B). Insets in the upper right corners display large pyramidal neurons in layer V (arrows). Scale bars, 1 mm in (A) and (B); 100 µm in the upper right corner insets.

**Figure 3:**
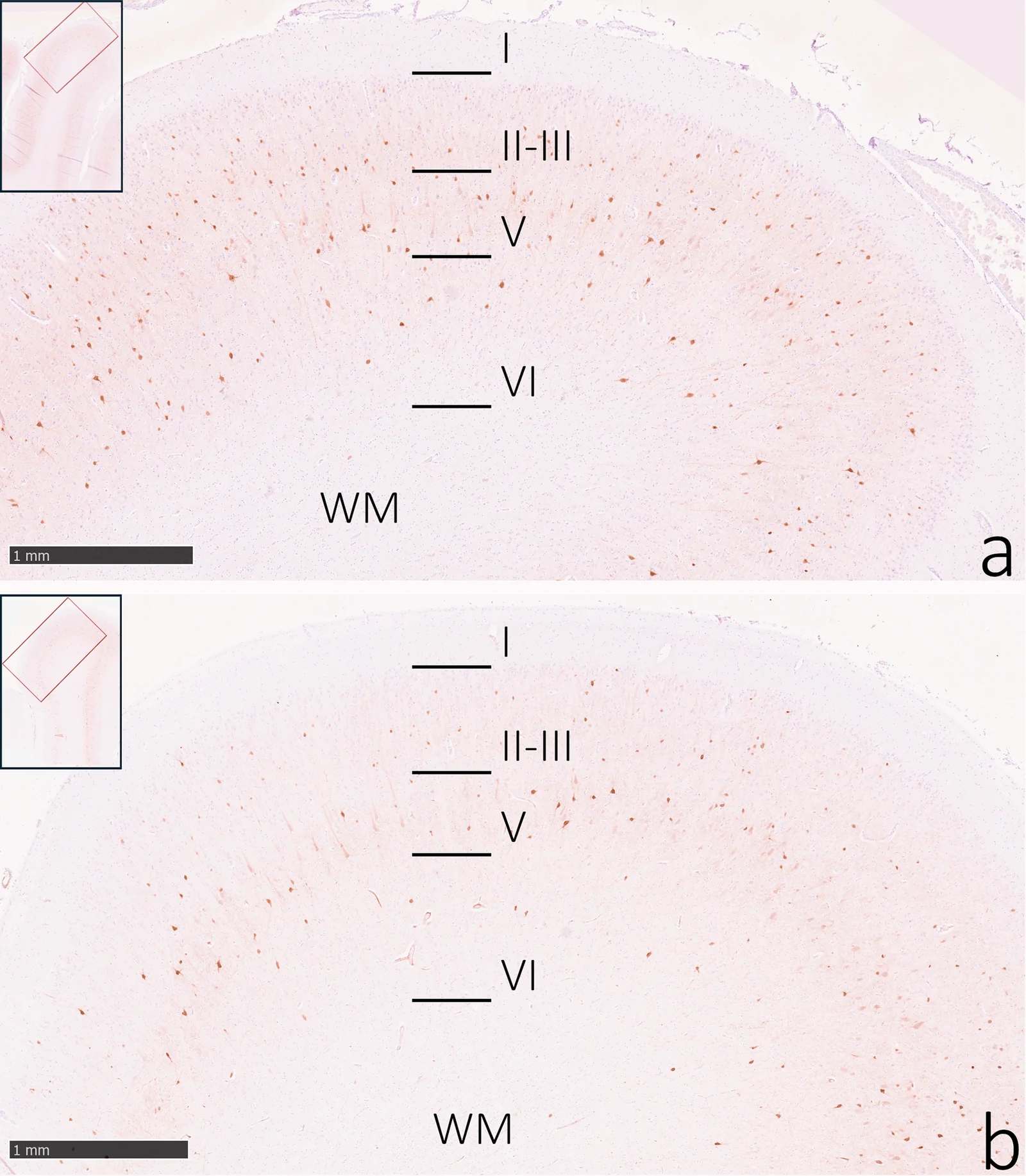
Distribution of parvalbumin-immunoreactive interneurons in M1. Immunostained sections showing parvalbumin-immunoreactive interneurons mainly localized in layers II–VI of the primary motor cortex in wild boars (A) and pigs (B). Insets in the upper left corners show the M1 gyrus, with red rectangles indicating the region in (A) and (B). Scale bars = 1 mm.

Nissl-stained coronal sections of the M1 in pigs and wild boars showed the typical laminar organization consisting of five distinct cortical layers, with layer IV nearly absent. Layer V was characterized by large pyramidal neurons (Figs. 2A, 2B). The distribution of parvalbumin-immunoreactive (PV-ir) interneurons did not reveal marked differences in laminar organization between pigs and wild boars. PV-ir cells were mainly distributed throughout layers II–VI in both species (Figs. 3A, 3B).

### Statistical analyses of morphological parameters

**Figure 4:**
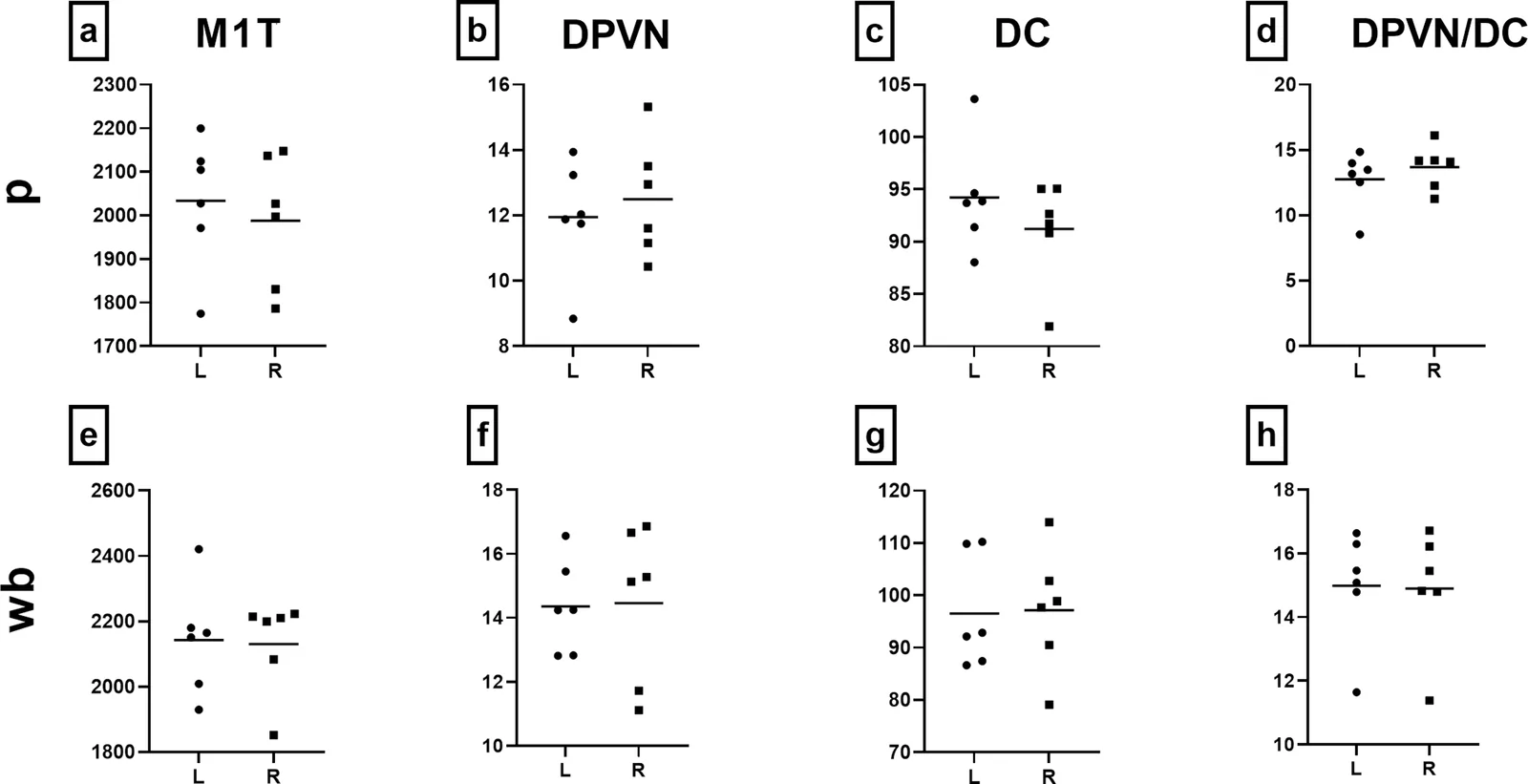
Comparative analysis of morphometric parameters in the left and right M1. Scatter plots showing the distribution of values for M1 thickness (M1T, µm), density of parvalbumin-immunoreactive neurons (DPVN, cells/mm^2^), cell density (DC, cells/mm^2^), and the DPVN/DC ratio in the primary motor cortex (M1) of the left (L) and right (R) hemispheres of pigs (p) and wild boars (wb). Each dot represents an individual animal. (A–D) and (E–H) compare the L and R hemispheres in pigs and wild boars, respectively (A–D; F–H: paired t-test; E: paired Wilcoxon test).

**Figure 5:**
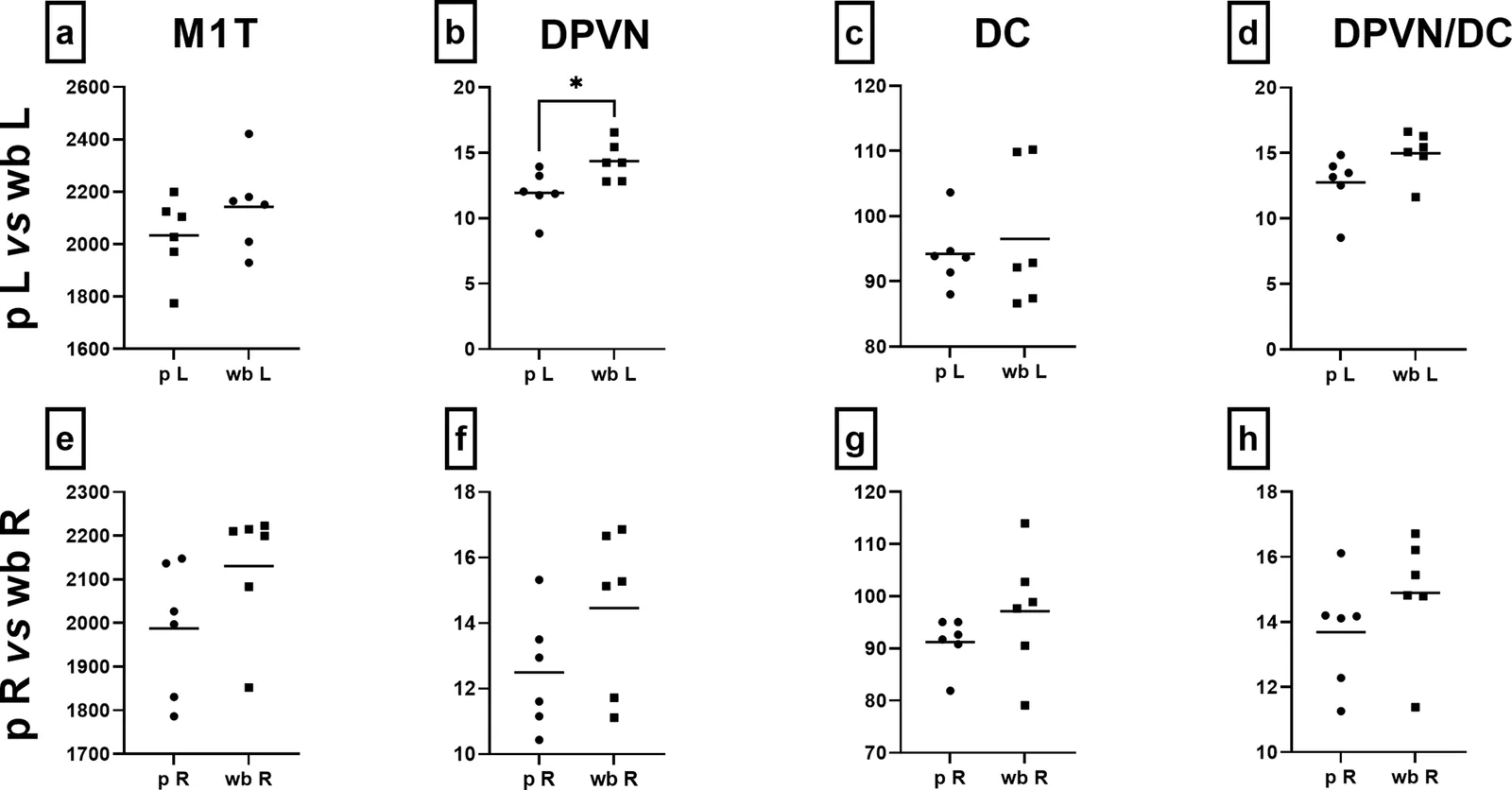
Comparative analysis of morphometric parameters across homologous M1 regions. Scatter plots showing the distribution of values for M1 thickness (M1T, µm), density of parvalbumin-immunoreactive neurons (DPVN, cells/mm^2^), cell density (DC, cells/mm^2^), and the DPVN/DC ratio in the primary motor cortex (M1) of the left (L) and right (R) hemispheres of pigs (p) and wild boars (wb). Each dot represents an individual animal. (A–D) and (E–H) show comparisons between the L hemispheres of pigs and wild boars, and between the R hemispheres, respectively (A–D, F–H: unpaired t-test; E: Mann–Whitney test). Asterisks indicate significance levels (*p < 0.05).

**Figure 6:**
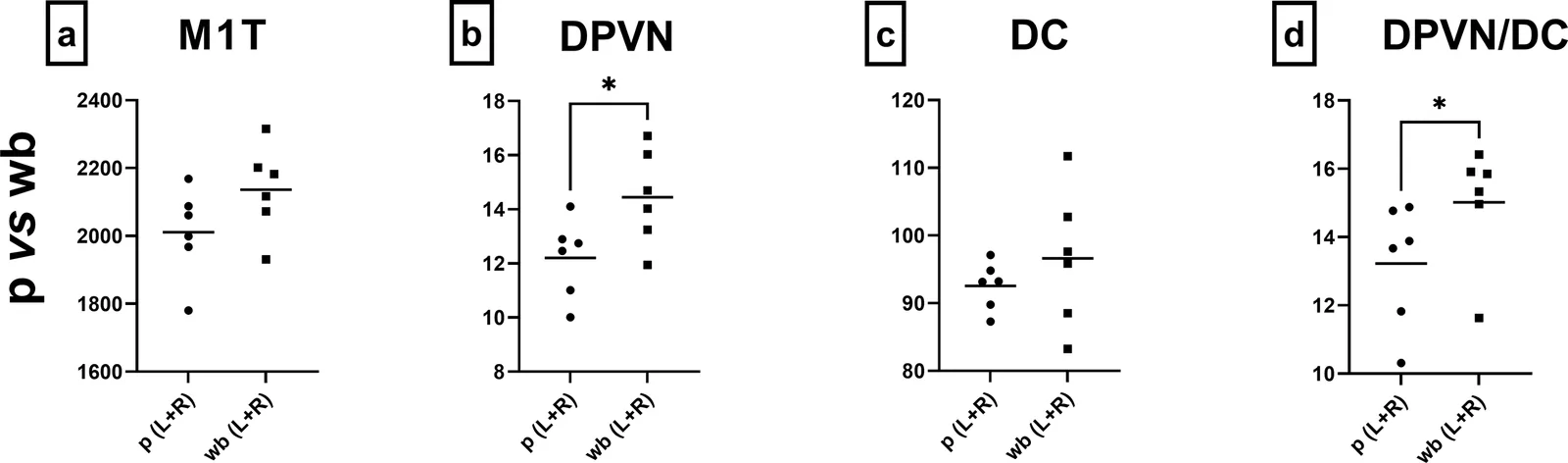
Comparative analysis of morphometric parameters across pooled left and right M1 regions. Scatter plots showing values for M1 thickness (M1T, µm), density of parvalbumin-immunoreactive neurons (DPVN, cells/mm^2^), cell density (DC, cells/mm^2^), and the DPVN/DC ratio in the primary motor cortex (M1) of the left (L) and right (R) hemispheres of pigs (p) and wild boars (wb). Each dot represents an individual animal. (A–D) illustrate comparisons between pooled hemispheres (L + R) of pigs and wild boars (A–C: unpaired t-test; D: Mann–Whitney test). Asterisks indicate significance levels (*p < 0.05).

Comparisons were performed for the following conditions: (1) pigs (p), left (L) *versus* (*vs*.) right (R) hemisphere; (2) wild boars (wb), L *vs*. R; (3) p L *vs*. wb L; (4) p R *vs*. wb R; and (5) p (L + R) *vs*. wb (L + R) (Figs. 4–6). Asterisks in the plots indicate statistically significant differences among datasets.

(1) Pigs L *vs*. R and (2) Wild boars L *vs*. R

No statistically significant differences were detected for M1T, DPVN, DC, or the DPVN/DC ratio between hemispheres for any of the four parameters analyzed (Figs. 4A–4H).

(3) Pigs L *vs*. wild boars L

The L DPVN of wild boars was higher than that of pigs in absolute value (Fig. 5B, *p* = 0.0273).

(4) Pigs R *vs*. wild boars R

No statistically significant differences were found for the measured parameters between the right hemisphere pig and wild boars.

(5) Pigs (L + R) *vs*. wild boars (L + R)

When hemispheres were combined, a higher DPVN both in absolute value (Fig. 6B, *p* = 0.0378) and relative to DC (Fig. 6D, *p* = 0, 0411) was observed in wild boars compared with pigs.

Effect size estimates (Table 2) revealed minimal hemispheric differences within species. In pigs, the comparison between left and right M1T yielded a small effect (*d* = 0.31), whereas in wild boars the corresponding effect was negligible (*d* = 0.08), indicating little to no lateralization in either group. In contrast, the comparison between species showed a large effect size (*d* = −0.96).

**Table 2:** Effect size analysis of cortical thickness.

Comparison	Cohen’s *d*	Interpretation
Pigs (left *vs*. right)	0.31	Small effect
Wild boars (left *vs*. right)	0.08	Negligible effect
Pigs *vs*. Wild boars	−0.96	Large effect

**Note:**

Cohen’s d indicates minimal hemispheric differences within species (pigs: 0.31; wild boars: 0.08) and a large effect between species (d = −0.96).

### Proteomic analysis

The efficiency of the extraction method applied to formalin-fixed, paraffin-embedded (FFPE) tissues was evaluated across both technical and biological replicates at the protein and peptide levels. The qualitative and quantitative reproducibility of protein extraction was assessed in terms of identification overlap (Venn diagrams) obtained from LC–MS/MS analysis (Figs. S4 and S5). Three technical replicates were analyzed for each sample, showing overlap values at the protein level ranging from 48.4% to 50.4% for pigs and 47.6% to 49.2% for wild boars. Across the three biological replicates, approximately 50% overlap in protein identification was observed between hemispheres for both species.

In the right (R) and left (L) pig cortices, the mean ± SD numbers of unique peptides and proteins identified were 3,264 ± 620 and 871 ± 145, and 3,487 ± 217 and 937 ± 53, respectively. For wild boars, the corresponding values were 3,066 ± 193 and 831 ± 33 for the R M1, and 3,106 ± 236 and 847 ± 64 for the L M1.

**Figure 7:**
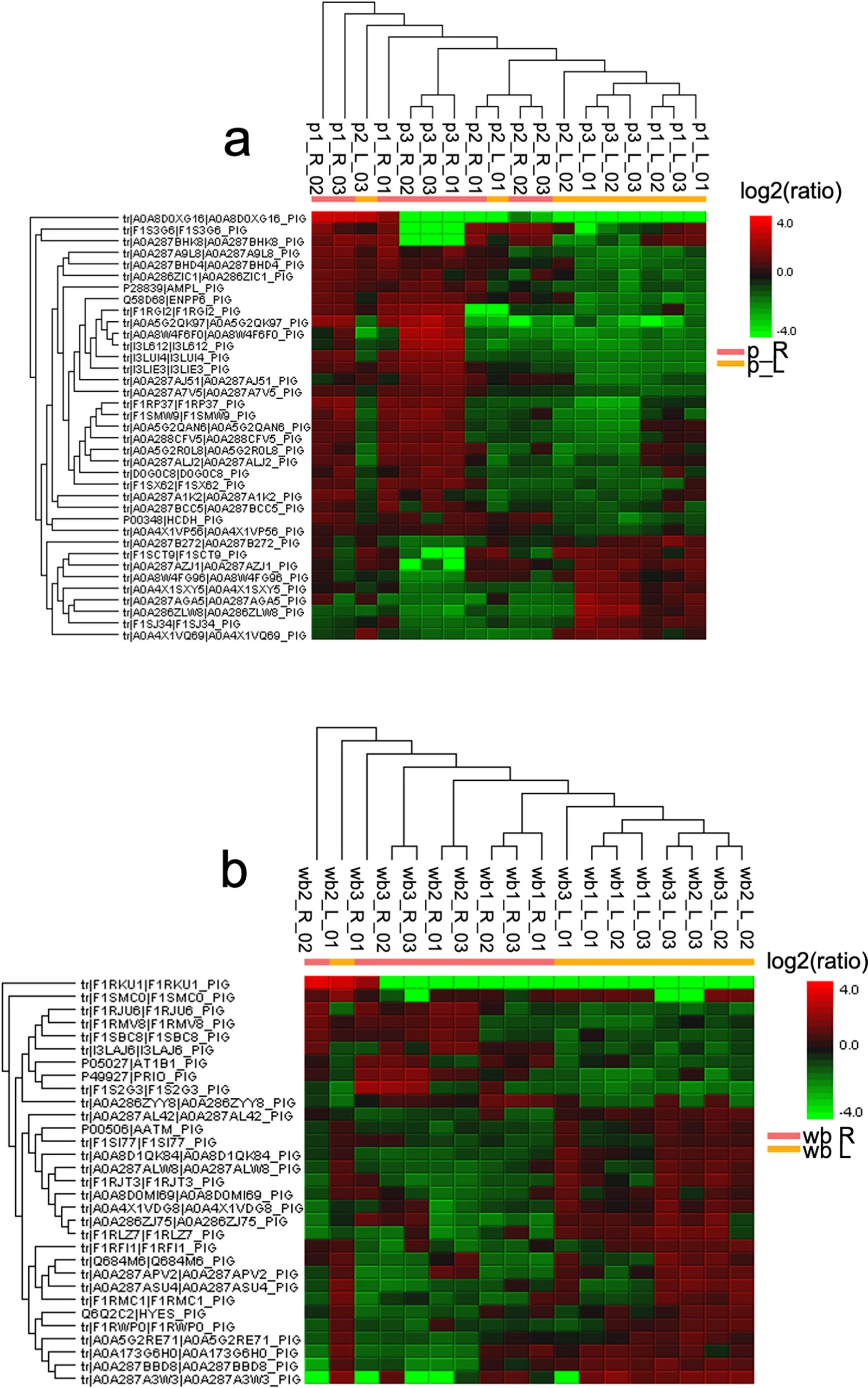
Proteomic comparison between left and right M1. Hierarchical clustering and heatmaps of Label-Free Quantitative (LFQ) raw intensity data obtained from comparisons between left (L) and right (R) primary motor cortex (M1) in pigs (A) and wild boars (B) analyzed using PEAKS Studio software. p, pig; wb, wild boar.

Initially, label-free quantitative (LFQ) data were compared to evaluate differences in protein expression between the R and L M1 within each species. The heatmaps summarize these differences for pigs (Fig. 7A) and wild boars (Fig. 7B). Thirty-two proteins were found to be differentially expressed (fold change ≥ 1.2) in pigs, 10 of which were upregulated in the L M1 compared to the R M1. In wild boars, 30 proteins were differentially expressed between L M1 and R M1, with 19 upregulated in the L M1. A complete list of identified proteins, including sequence coverage, number of unique peptides, and MS/MS parameters, is provided in Tables S1 and S2.

Of particular note, the astrocytic phosphoprotein PEA-15 and pyrroline-5-carboxylate reductase 3 (PYCR3) showed increased expression in the L M1—by 1.6-fold in pigs and 1.4-fold in wild boars. Both proteins are associated with myelination processes.

**Figure 8:**
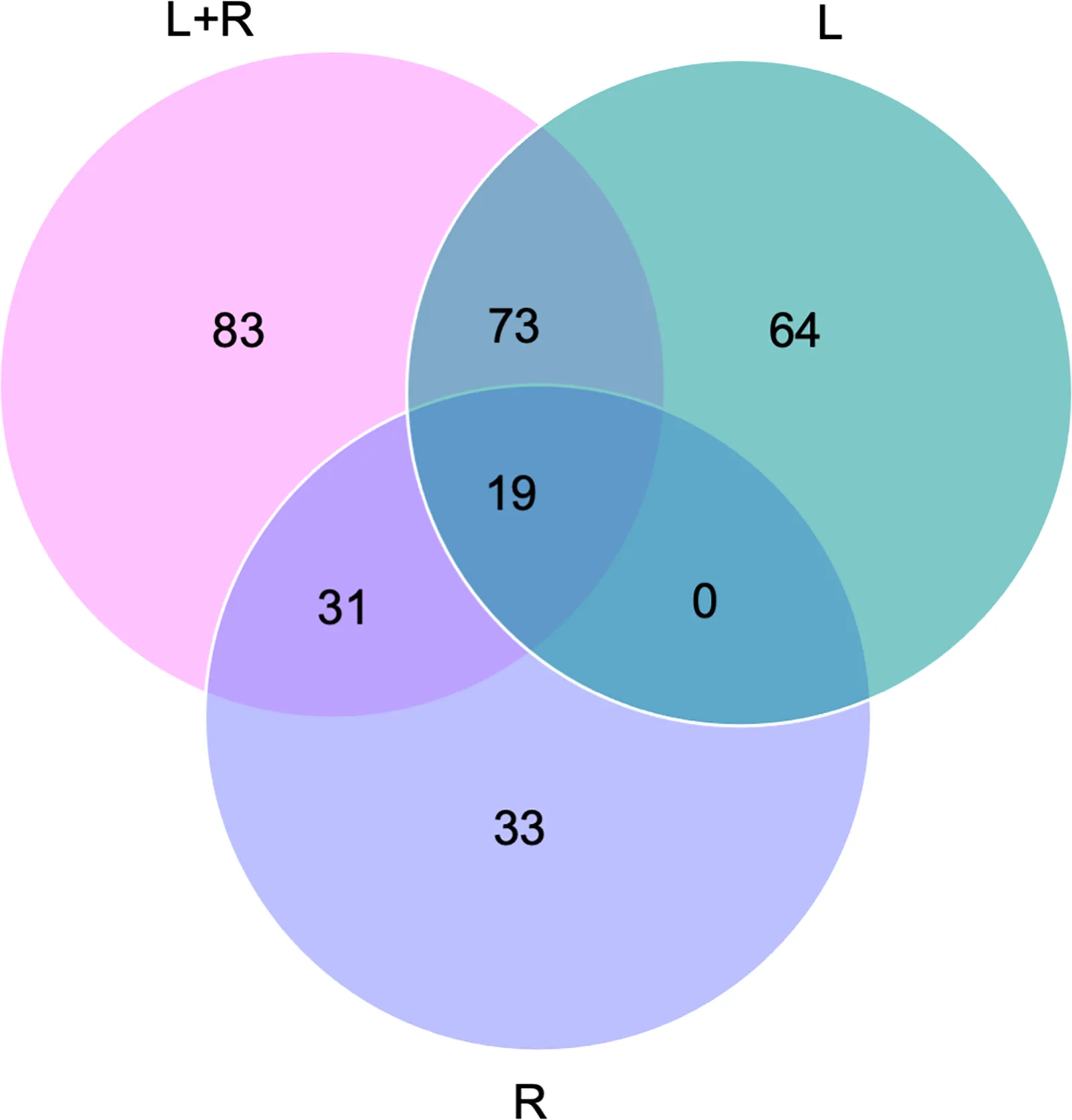
Shared and unique differentially expressed proteins between pigs and wild boars. Venn diagram showing the overlap of differentially expressed proteins identified in the various comparisons between wild boar and pig primary motor cortex (M1). L, left hemisphere; R, right hemisphere.

To investigate potential molecular correlates of domestication, quantitative data from shotgun proteomic analysis of M1 samples were compared between species, both by hemisphere (wild boar L *vs*. pig L; wild boar R *vs*. pig R) and in combined form (wild boar L + R *vs*. pig L + R). The Venn diagram in Fig. 8 illustrates the overlap of differentially expressed proteins across the three comparisons. Only proteins showing a fold change ≥ 1.2 were considered. Of the 303 proteins analyzed, 19 were common to all comparisons, while 27%, 11%, and 21% were unique to the L + R, R, and L comparisons, respectively.

**Figure 9:**
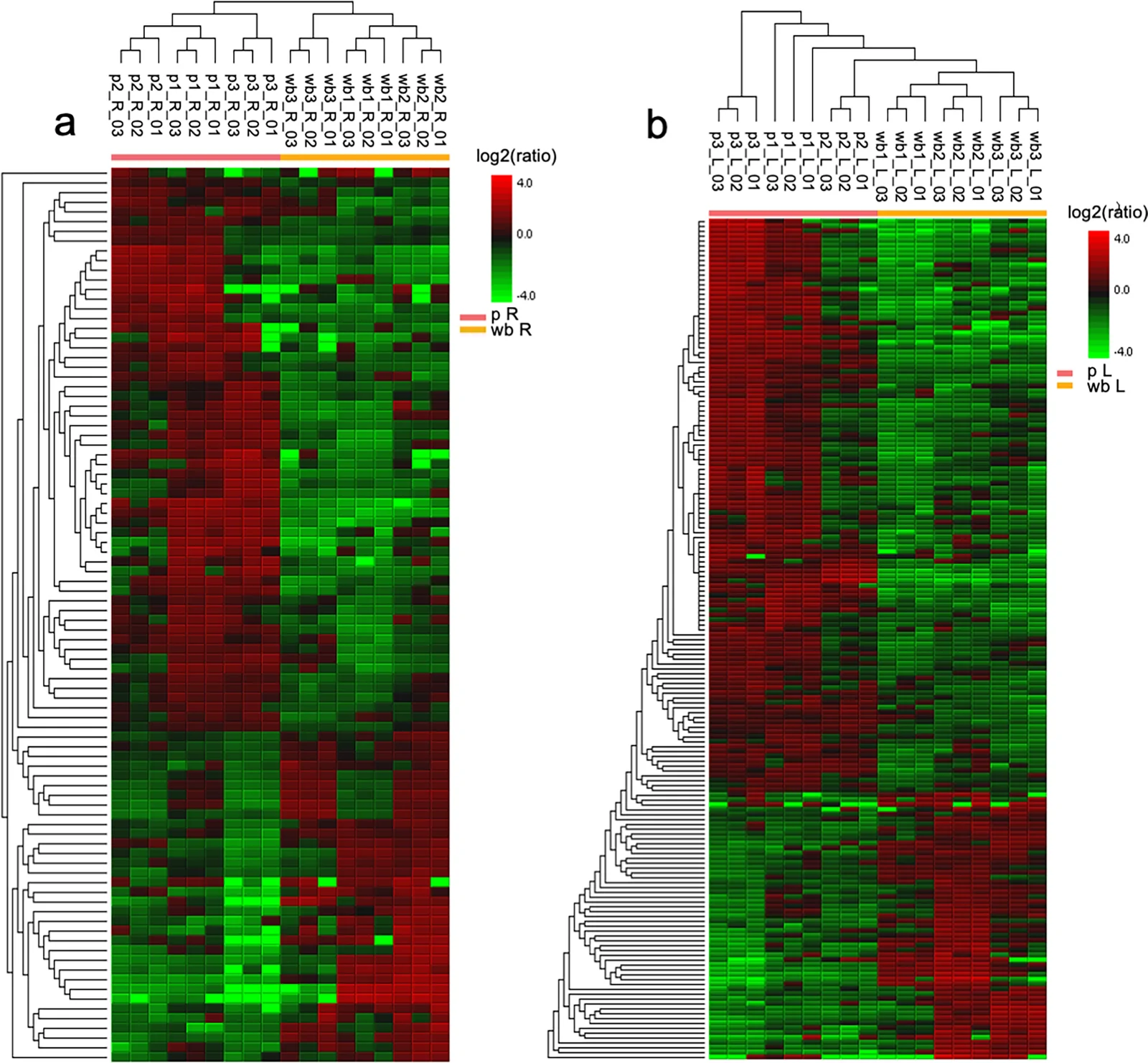
Proteomic comparison between homologous M1 regions. Hierarchical clustering and heatmaps of Label-Free Quantitative (LFQ) raw intensity data. (A) Comparison of the right (R) primary motor cortex (M1) of wild boars (wb) with the R M1 of pigs (p). (B) Comparison of the left (L) primary motor cortex (M1) of wild boars (wb) with the L M1 of pigs. Data were analyzed using PEAKS Studio software.

**Figure 10:**
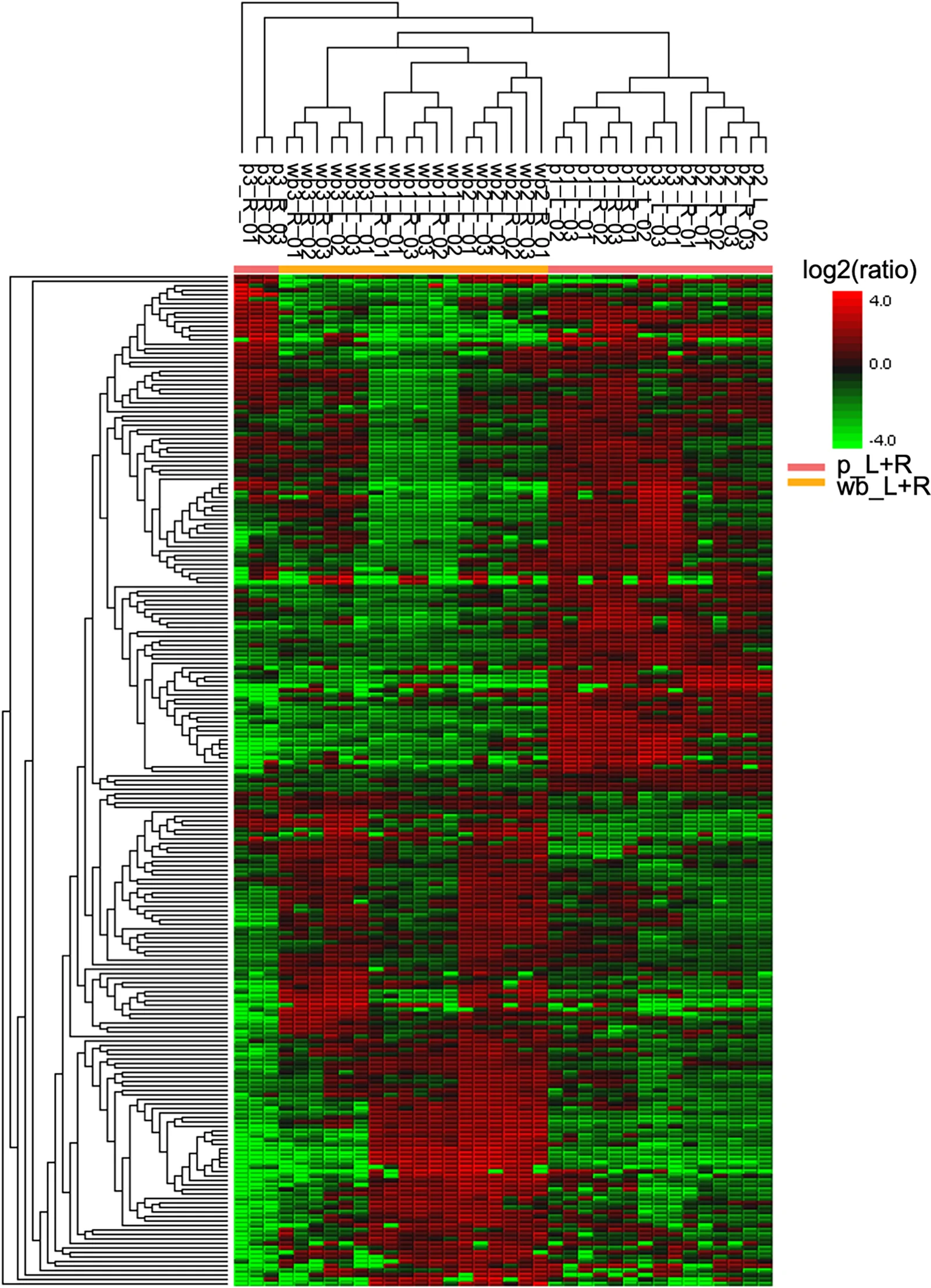
Proteomic comparison of pooled left and right M1 regions. Hierarchical clustering and heat maps of Label-Free Quantitative (LFQ) raw intensity data. Shown is a comparison of the left (L) and right (R) primary motor cortex (M1), pooled together (L + R), between wild boars (wb) and pigs (p).

As shown in the heatmaps (Figs. 9, 10), both the technical and biological replicates clustered closely within each species, indicating high internal consistency. In the L + R comparison (Fig. 10), 205 proteins were differentially expressed, 108 of which were overexpressed in wild boars relative to pigs (fold change ≥ 1.2). A detailed list of these proteins, along with MS parameters and expression ratios, is provided in Table S4.

STRING enrichment analysis of all proteins from this comparison revealed that the main biological processes involved included gliogenesis, glial cell differentiation and development, myelination, regulation of synaptic plasticity, dopamine receptor signaling, and modulation of chemical synaptic transmission, all with false discovery rate (FDR) < 0.002 (Fig. S6).

Separate STRING analyses were then conducted for proteins overexpressed in each species. In wild boars, upregulated proteins were primarily associated with the dopamine receptor signaling pathway, cellular response to dopamine, and metabolic processes such as ribonucleoside diphosphate metabolism and small-molecule processing. In contrast, in pigs, upregulated proteins were enriched in processes related to the modulation of chemical synaptic transmission, exocytosis, and cellular secretion (Figs. S7, S8).

Among the proteins overexpressed in wild boars, notable examples included dynamin GTPase (DCM1), glutathione peroxidase (GPx1), G protein subunit z (Gαz), and synaptotagmin-12 (SYT12), which showed increases ranging from 2.0- to 4.1-fold. Conversely, lower expression levels were observed for glial fibrillary acidic protein (GFAP) and myelin basic protein (MBP) in wild boars compared with pigs. Comparative analyses of the R and L M1 separately (Figs. 9A, 9B) revealed that, out of 156 proteins, 55 were overexpressed in wild boars relative to pigs in the L M1. In the R M1, 83 proteins were differentially expressed, of these 35 upregulated in wild boars. Table S4 provides full details of the proteins, mass spectrometry data, and expression ratios for the L and R comparisons, respectively.

## Discussion

In this study, we conducted a microscopic and molecular comparison of the M1 in pigs and wild boars to evaluate potential effects of domestication. The macroscopic identification of M1, based on the position of the *sulcus coronalis, sulcus cruciatus*, and *sulcus ansatus*, was confirmed by histological analysis. In Nissl-stained sections, M1 was defined according to two well-established anatomical markers (Yamawaki et al., 2014; Zilles, 2018): the presence of large pyramidal neurons in layer V and the near absence of cortical layer IV. The observed cytoarchitecture was consistent with that previously described in Göttingen minipigs (Bjarkam et al., 2017), as well as in other ungulates and cetaceans (Hof, Chanis & Marino, 2005; Hof & Gucht, 2007; Cozzi et al., 2017; Peruffo et al., 2019).

In our sampling of M1, however, we could not account for the intrinsic complexity that may vary across different motor representations (*e.g*., face *vs*. limb areas). This organizational feature has been extensively described in the domestic pig, in which M1 gives rise to corticospinal and brainstem projections, indicating a structured motor territory consistent with somatotopic control (Breazile, Swafford & Thompson, 1966; del Cerro, Rodríguez-De-Lope & Collazos-Castro, 2021). Consequently, the lack of representation-specific sampling within M1 may have limited our ability to detect potential region-dependent differences in cortical organization.

### Immunohistochemistry

In the present study, the quantification of parvalbumin-positive interneurons in M1 was not performed using a design-based stereological approach. Design-based stereology is widely regarded as the standard for obtaining assumption-free and unbiased estimates of neuronal populations (West, Slomianka & Gundersen, 1991; Schmitz & Hof, 2005). Accordingly, the differences reported here should be interpreted as relative differences within the sampled regions rather than definitive estimates of total parvalbumin interneuron number or density across M1.

Immunohistochemical analysis showed that parvalbumin-immunoreactive (PV-ir) interneurons were mainly distributed in layers II–VI, confirming previous observations in adult and juvenile pigs (Desantis et al., 2021). A similar distribution pattern has also been described in the M1 of non-human primates, including chimpanzees and macaques (Cozzi et al., 2017), and in the somatosensory cortex of rodents such as mice, rats, and gerbils (Ahn et al., 2017).

DPVN and DPVN/DC values indicated that the left hemisphere was the principal contributor to the elevated measures observed in wild boars. Pigs exhibited lower DPVN and DPVN/DC values, suggesting that domestication may have influenced the cytoarchitecture of M1. Although not statistically significant, M1T tended to be greater in wild boars than in pigs, with a large effect size supporting this trend; this observation may warrant further investigation in larger cohorts.

In pigs, the M1 and premotor cortex regulate spinal circuits both directly *via* the corticospinal tract and indirectly through the brainstem. Cervical spinal neurons control forelimb movements, whereas hindlimb movements are mediated by brainstem pathways (del Cerro, Rodríguez-De-Lope & Collazos-Castro, 2021). Wild boars exhibited a higher density of PV-ir interneurons in M1 which may support more refined motor control critical for survival in natural environments. This interpretation aligns with the role of PV-expressing interneurons, which modulate the excitatory output of pyramidal neurons and are key regulators of voluntary movement (Estebanez et al., 2017).

The M1 and cerebellum represent the principal structures involved in generating and refining motor output (Apps & Garwicz, 2005; Ebbesen & Brecht, 2017). In a previous microscopic-scale study, we demonstrated a higher linear density of Purkinje cells in the cerebellum of wild boars compared with pigs (Pirone et al., 2022). Together, the present M1 findings, considered alongside our previous cerebellar data, raise the possibility that pigs may differ in aspects of motor control organization. Notably, both the present results on PV interneurons and our earlier data on Purkinje cells (Pirone et al., 2022) derive from structures within the same brain, reinforcing this interpretation. However, in the absence of direct functional or behavioral measurements, this interpretation remains provisional.

Furthermore, our findings suggest that similar to the excitatory glutamatergic system (Hecht et al., 2023), the inhibitory GABAergic network, particularly PV-ir interneurons, may also be influenced by domestication. These two systems are functionally interdependent, and their balance is crucial for normal cortical processing. Dysfunctions of PV interneurons disrupt excitatory/inhibitory homeostasis within cortical circuits and have been implicated in various neurological disorders (Leitch, 2024).

Nevertheless, the inhibitory GABAergic network may also be susceptible to alterations induced by transport and handling. In this context, studies on pigs reared under intensive farming conditions have demonstrated that stress exposure can modulate their neurophysiology, producing region-specific changes in monoaminergic systems (5-HT, DA, NA) within the amygdala, hippocampus, hypothalamus, and prefrontal cortex (Martínez-Miró et al., 2016; Arroyo et al., 2019). Moreover, environmental enrichment has been associated with structural and molecular adaptations in the cortex, including effects on parvalbumin-positive interneurons (Kempermann, Kuhn & Gage, 1997; Serra et al., 2020; Terstege & Epp, 2026). These findings highlight the sensitivity of cortical and subcortical inhibitory networks to environmental and management factors, which may secondarily influence higher-order cognitive and emotional regulation.

Furthermore, although we were unable to assess the effect of sex, it may have contributed to the differences observed in M1. While no significant sex differences in overall brain size between wild boars and domestic pigs have been reported (Cucchi et al., 2024), sex-linked differences have been described in porcine brain. For example, Conrad, Dilger & Johnson (2012) reported sex-dependent differences in brain growth trajectories, and during development certain hypothalamic nuclei exhibit a greater number of neurons in females (Van Eerdenburg & Swaab, 1994). In addition, in the fetal pig brain, neuronal and astrocytic gene markers are differentially expressed between the sexes (Strawn et al., 2021).

Because neural architecture is tightly linked to higher-order brain functions (White, 2007), behavioral differences between wild and domesticated species may be partly explained by structural and physiological variations in the central nervous system (CNS).

Macroscopic evidence indicates that domesticated species generally exhibit smaller brains than their wild progenitors (Castiglione et al., 2020; Balcarcel et al., 2022). In pigs, Kruska (2005) reported a 34% reduction in the brain-to-body mass ratio relative to wild boar. Using an allometric regression of brain mass or endocranial volume (EV) against body size, Balcarcel et al. (2022) estimated a 24% reduction, whereas Cucchi et al. (2024), based on endocast volume measurements, reported a reduction of approximately 18%.

In the present study, body weight data were incomplete; however, brain weight was assessed. Absolute brain size, rather than body size or encephalization quotient, has been proposed as a more reliable parameter for assessing cognitive abilities across species (Deaner et al., 2007).

Although our data report absolute brain weight, the magnitude of the difference observed in pigs appears consistent with that described in the previously cited studies (Kruska, 2005; Balcarcel et al., 2022; Cucchi et al., 2024) and paralleled the decrease in DPVN, while DC remained unchanged. Though brain weight differed between groups, brain mass could not be interpreted as a direct proxy for neuronal density. Across mammals, increases in brain size have not been linearly associated with increases in neuronal packing density, as brain mass reflects multiple structural components, including white matter expansion, glial content, and neuropil complexity (Herculano-Houzel & Lent, 2005; Herculano-Houzel, 2012). Therefore, we speculate that, in pigs, factors other than PV interneurons may have contributed to the observed reduction in brain weight.

Morphofunctional alterations related to domestication have also been described in the CNS of other species, including sheep (Ebinger, 1975), dogs (Grewal et al., 2020), and rabbits (Brusini et al., 2018). The cognitive advantages associated with larger brain size in wild animals likely reflect enhanced spatial memory, navigation ability, behavioral flexibility, and complex foraging strategies for acquiring nutrient-rich resources (Sol et al., 2008; Benson-Amram et al., 2016; Schuppli et al., 2016; Powell, Isler & Barton, 2017).

### Proteomic analysis

To explore molecular changes potentially associated with the domestication process, we performed a deep proteomic analysis of the M1 in pigs and wild boars. The analysis was conducted on formalin-fixed, paraffin-embedded (FFPE) tissues using a gel-free approach, confirming the reproducibility and reliability of the applied methodology (Donadio et al., 2011). However, the limited biological replication (*n* = 3 per group) represents a potential limitation, as it reduces statistical power and may affect the robustness and generalizability of differential expression results; therefore, these findings should be interpreted with caution and warrant validation in studies including a greater number of animals.

A large number of proteins and peptides were identified with high-quality mass spectrometric parameters. Comparative analyses of label-free quantitative (LFQ) data were carried out to identify significant differences in protein expression, aiming to assess possible cortical asymmetry within each species and potential functional differences between wild boars and pigs.

Comparisons between the L and R M1 revealed only limited differences; however, in both species, increased expression of proteins associated with myelination was observed in the L hemisphere, suggesting enhanced glial cell activity on that side. In pigs, PEA-15 was overexpressed in the L M1. This protein promotes cell viability, reduces apoptosis, and contributes to the development and organization of the cerebral cortex and white matter tracts (Xian, Li & Chen, 2019; Graff et al., 2020).

In wild boars, a significant upregulation of pyrroline-5-carboxylate reductase 3 (PYCR3) was detected. Similar to its mitochondrial isoforms, PYCR3 facilitates oligodendrocyte differentiation and myelin formation, providing protection for axons against metabolic and mechanical stress (Torii et al., 2022). This finding may be consistent with the trend toward greater M1 thickness in wild boars, possibly reflecting differences in glial content and neuropil complexity (Stiles & Jernigan, 2010). Although this interpretation would require confirmation through targeted analyses.

Wild boars also exhibited a higher density of PV-ir interneurons which play a key role in modulating cortical motor output that is essential for rapid and adaptive responses in natural environments. This hypothesis is further supported by proteomic data showing overexpression of proteins involved in oxidative stress defense and mitochondrial maintenance in the wild boar M1. PV-interneurons are characterized by fast-spiking activity that demands substantial bioenergetic resources (Jiang, Cowell & Nakazawa, 2013; Pinna & Colasanti, 2021). High metabolic activity leads to increased production of reactive oxygen species (ROS) during mitochondrial respiration, rendering these neurons particularly vulnerable to oxidative stress (Wang et al., 2024).

Consistent with the prominent PV-ir network in wild boar M1, we observed elevated expression of proteins related to oxidative stress protection, including glutathione peroxidase (GPx1), and of those involved in maintaining mitochondrial morphology, such as dynamin-related proteins (DCM3 and DCM1). GPx1 plays a key role in detoxifying hydrogen peroxide *via* the glutathione (GSH) pathway, thereby limiting ROS accumulation. Reduced GPx1 activity, conversely, enhances mitochondrial fission (Sun et al., 2020; Yang et al., 2023) and exacerbates oxidative stress in PV-interneurons, leading to impaired PV expression (Cabungcal et al., 2013). The preservation of mitochondrial morphology and volume during sustained neuronal firing relies on proteins such as DCM3 and DCM1, which stabilize mitochondrial dynamics and prevent excessive fission (Lichvarova et al., 2018). Similarly, the overexpression of carbonyl reductase (NADPH) may reflect an additional antioxidant response, as this enzyme neutralizes reactive carbonyl species generated by oxidative stress (Oppermann, 2007).

In addition to stress-protective mechanisms, the wild boar M1 exhibited upregulation of synaptotagmin-12 (SYT12) and several proteins involved in the dopamine receptor signaling pathway—both functionally associated with synaptic plasticity (Speranza et al., 2021). Although the role of dopamine in interspecific neurochemical variation during domestication remains unclear, dopamine is known to modulate synaptic plasticity in the primary motor cortex during practice-dependent motor learning (Wolfes & Dean, 2020; Macedo-Lima & Remage-Healey, 2021). Moreover, in the L M1 of wild boars, we detected a significant increase in dihydropyrimidinase-like (DPYSL) proteins, which participate in intracellular and extracellular signaling and play key roles in neuronal processes including cell migration, neurite extension, axonal guidance, dendritic spine formation, and synaptic remodeling (Desprez et al., 2023).

Overall, the proteins overexpressed in wild boar suggest a potential enhancement of pathways involved in myelination, oxidative stress regulation, and synaptic plasticity. However, we cannot exclude the possibility that this pattern may be influenced by environmental enrichment (Stein et al., 2016; Gao et al., 2022; Ramos et al., 2024), rather than by domestication, as this issue remains a matter of debate (Hecht et al., 2023).

Behavioral and neurological adaptations associated with domestication likely depend on modifications in synaptogenesis and network plasticity, which underpin neural communication and adaptability to new social and environmental conditions (Hecht et al., 2023). The interplay between neuroplasticity and adaptability thus provides an intriguing framework for understanding how cortical circuits evolve under domestication-related selective pressures.

### Limitations

This study has several limitations. One relates to the sampling strategy for the motor cortex: to obtain representative tissue from the M1, two blocks were collected from each hemisphere approximately 1 cm apart. Although this approach allowed for anatomical consistency, it was not stereologically designed and therefore may limit the quantitative accuracy of the estimates. In addition, in calculating DC, only cells with a clearly visible nucleus were counted, which could have resulted in a slight underestimation of total cell number.

A further limitation concerns the absence of sex-specific data for pigs, which precluded the evaluation of possible sex-related effects of domestication. Although a recent study reported no significant sex differences in brain size between wild boars and domestic pigs (Cucchi et al., 2024), the potential influence of sex on neural architecture remains debated and warrants further investigation (Grewal et al., 2020).

Another constraint lies in the use of FFPE tissue for proteomic analysis. Proteins in FFPE samples undergo chemical modifications during fixation and embedding, and analyses of fresh, unfixed material generally yield higher-quality proteomic profiles. Nonetheless, previous studies have demonstrated that reliable proteomic data can be obtained from FFPE samples (Addis et al., 2009; Donadio et al., 2011; Kawashima et al., 2014; Davalieva et al., 2021). Moreover, the use of archived embedded material enables retrospective studies and, importantly as in the present work, avoids the need to sacrifice additional animals.

## Conclusions

The findings of this study suggest that both the cytoarchitecture and protein profile of the pig M1 may have been reshaped during the domestication process. Particularly noteworthy are the reductions in PV-ir interneurons in the M1 and in Purkinje cells previously reported in the cerebellum, which together point to modifications in neuronal network properties in domestic pigs.

Collectively, these results suggest that the wild boar may exhibit more finely tuned regulation of motor control, supported by enhanced mechanisms to sustain neuronal activity and viability. However, domestication is a complex and multifactorial process influenced by genetic, environmental, developmental, and selective pressures. Further investigations integrating morphological, molecular, functional, and behavioural approaches, and including a greater number of animals, are necessary to better elucidate the mechanisms underlying brain modifications associated with domestication.

## Supplemental Information

10.7717/peerj.21640/supp-1 Supplemental Information 1 Histological raw data of the M1 measured parameters.

10.7717/peerj.21640/supp-2 Supplemental Information 2 Brain Weight in Domestic Pigs and Wild Boars. Brain weight (expressed in grams) measured in individual animals belonging to two groups: domestic pigs (Pigs) and wild boars (Wild boars). Each animal is identified by a specific code (Animal ID).

10.7717/peerj.21640/supp-3 Supplemental Information 3 Antibodies. The primary and secondary antibodies used in the study, including their immunogen, supplier details, catalog and RRID numbers, and working dilution.

10.7717/peerj.21640/supp-4 Supplemental Information 4 The quantitative extracted morphometric parameters for each set of images acquired.

10.7717/peerj.21640/supp-5 Supplemental Information 5 Proteomic raw data. Raw data showing the list of protein identifications obtained by comparing the left (L) and right (R) hemispheres of the cortex in pig (p) and wild boar (wb). The significance of the differences was assessed by ANOVA using the following protein-level thresholds: −10 lgP ≥ 20, fold change ≥ 1.2, and at least one peptide used for quantification. Protein amounts were normalized to cytoplasmic actin 1 (Q6QAQ1-ACTB_PIG).

10.7717/peerj.21640/supp-6 Supplemental Information 6 Supplementary materials on histology, immunohistochemistry and proteomic analyses.

## Additional Information and Declarations

### Data Availability

The following information was supplied regarding data availability:

The raw data is available in the Supplemental Files.

The proteomics data are accessible *via* the Massive repository:

- https://massive.ucsd.edu/ProteoSAFe/private-dataset.jsp?task=125b2e5973ce4c3689255fd77d6a0fc1.
