# Supplementary material for: Comparative cytoarchitectural and proteomic analysis of the primary motor cortex in pigs and wild boars reveals domestication associated changes
Source: PeerJ. 2026 Aug 18;14:e21640. doi: 10.7717/peerj.21640 (PMC13499532; doi:10.7717/peerj.21640)
Supplement: Supplementary file 1 [file peerj-14-21640-s006.pdf]

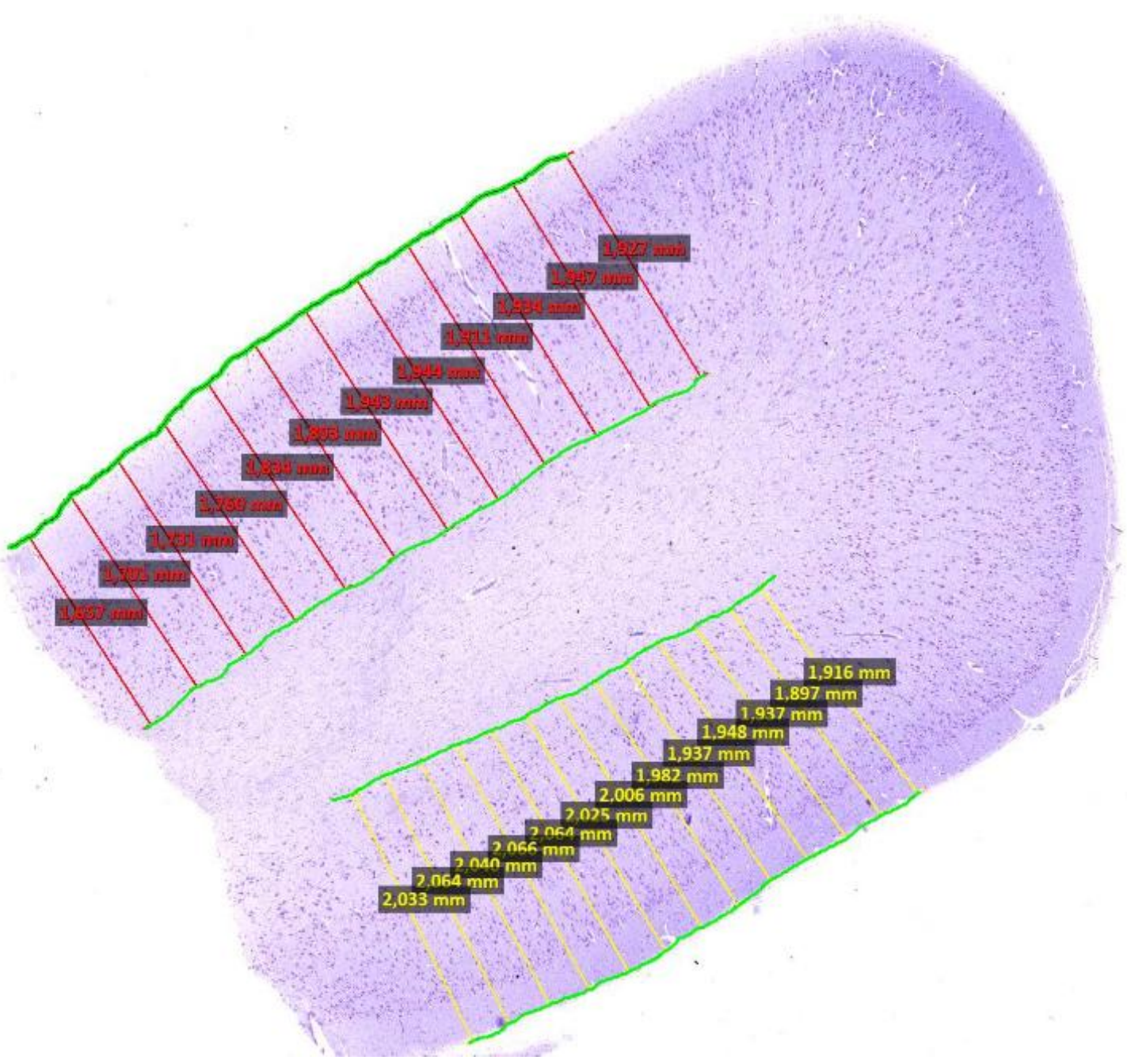


Figure 1. Measurement of the M1 thickness was conducted on a transversely cut Nissl-stained section of the motor cortex. Two green lines, representing the pial surface and the gray matter (gm)/white matter (wm) interface, were manually drawn on both sides of the gyrus. The ImageScope software then automatically measured the distance by placing 25 perpendicular segments connecting the two lines.

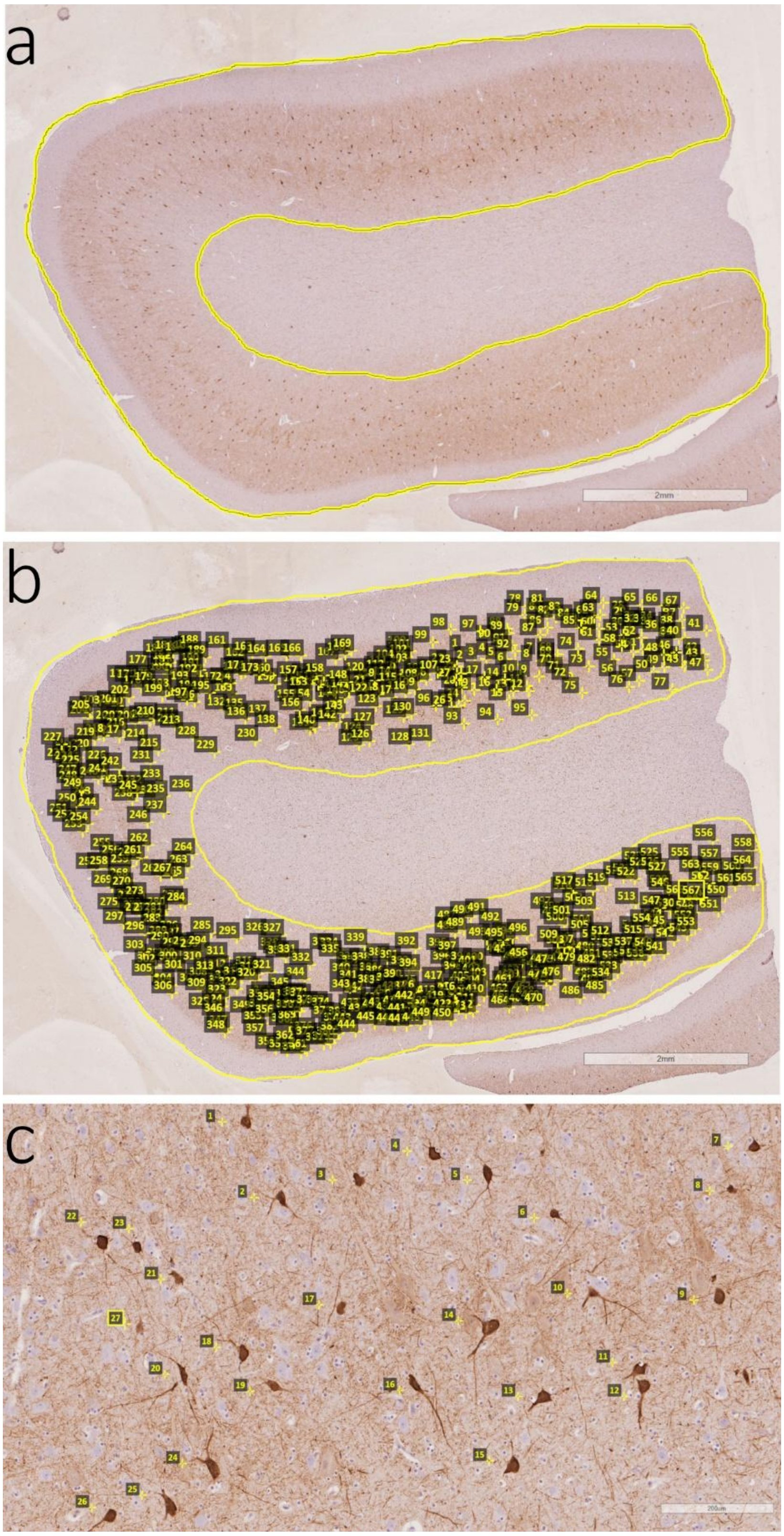


Figure 2. Evaluation of the density of PV-ir neurons in the motor cortex. The ROI in PV immunostained coronal sections was manually traced (a) and all the PV-ir somata were counted (b). The Image Scope software assigned a label to avoid counting the same object twice (c). Bars in (a) and (b) are 2 mm, in (c) is 200 mm.

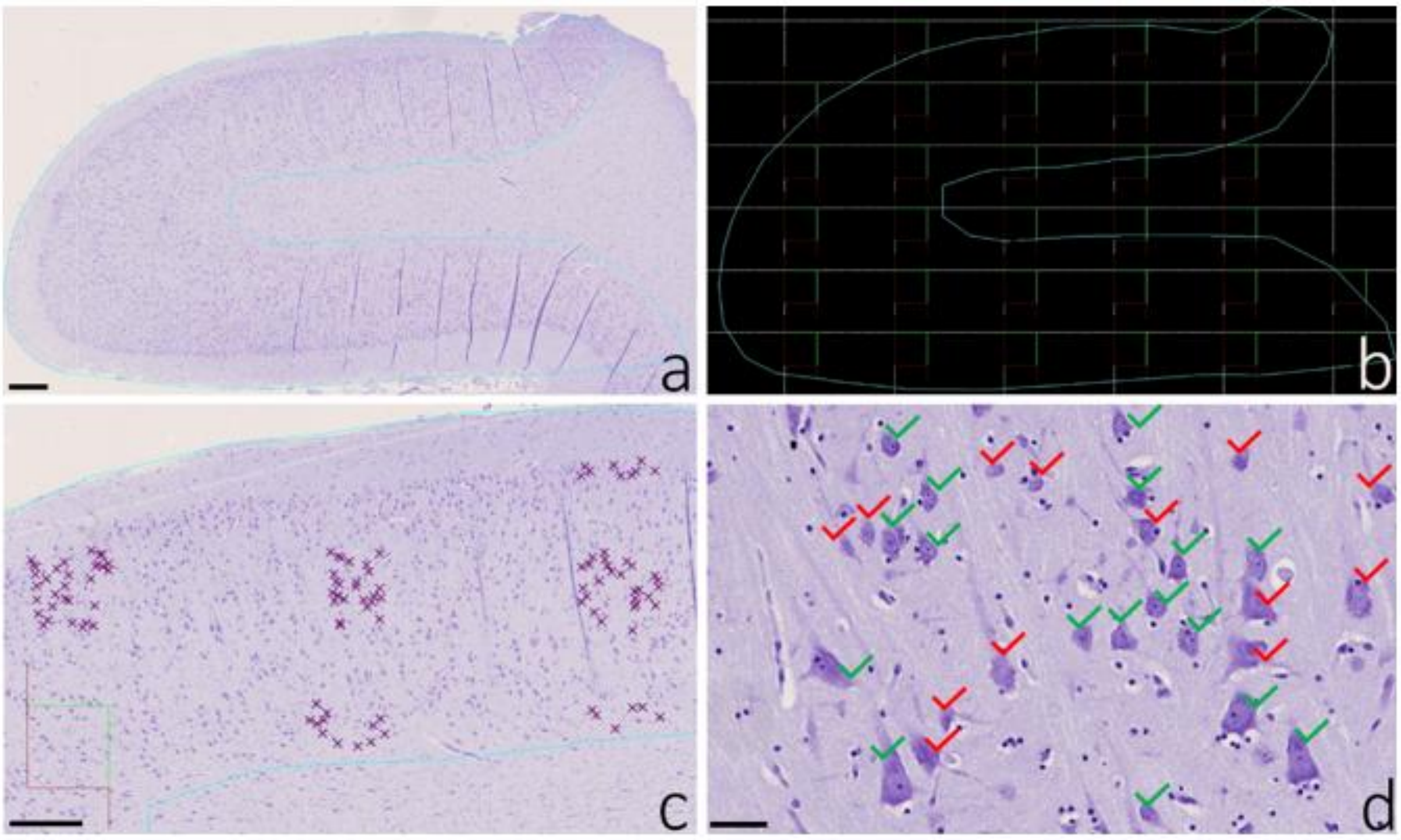


Figure 3. Evaluation of Cell Density. The region of interest (ROI), extending from the pial surface to the gm/wm interface, was traced using the contour tool (a). The software placed probes in a systematic random sampling grid overlaid onto the ROI (b), and cells within the counting frame (red and green square above the scale bar) were counted (c). Only cells with a visible nucleus were considered (d, green ticks indicate counted cells, red ticks indicate excluded cells). The bars are 500 µm in (a) and (c), and 50 µm in (d).

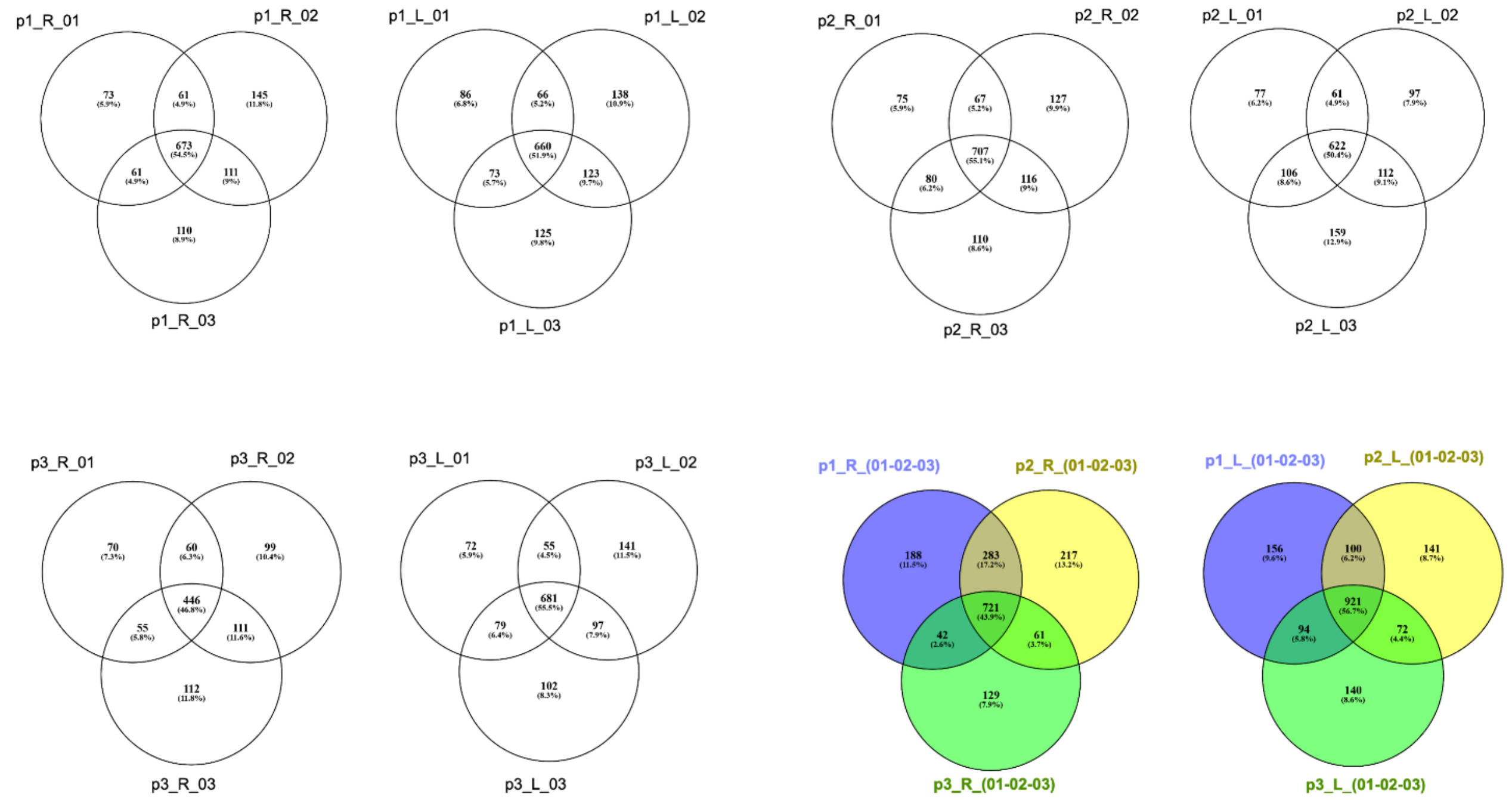


Figure 4. Venn diagrams showing overlap of protein identifications obtained in technical and biological replicates from pig samples. L, left; R, right.

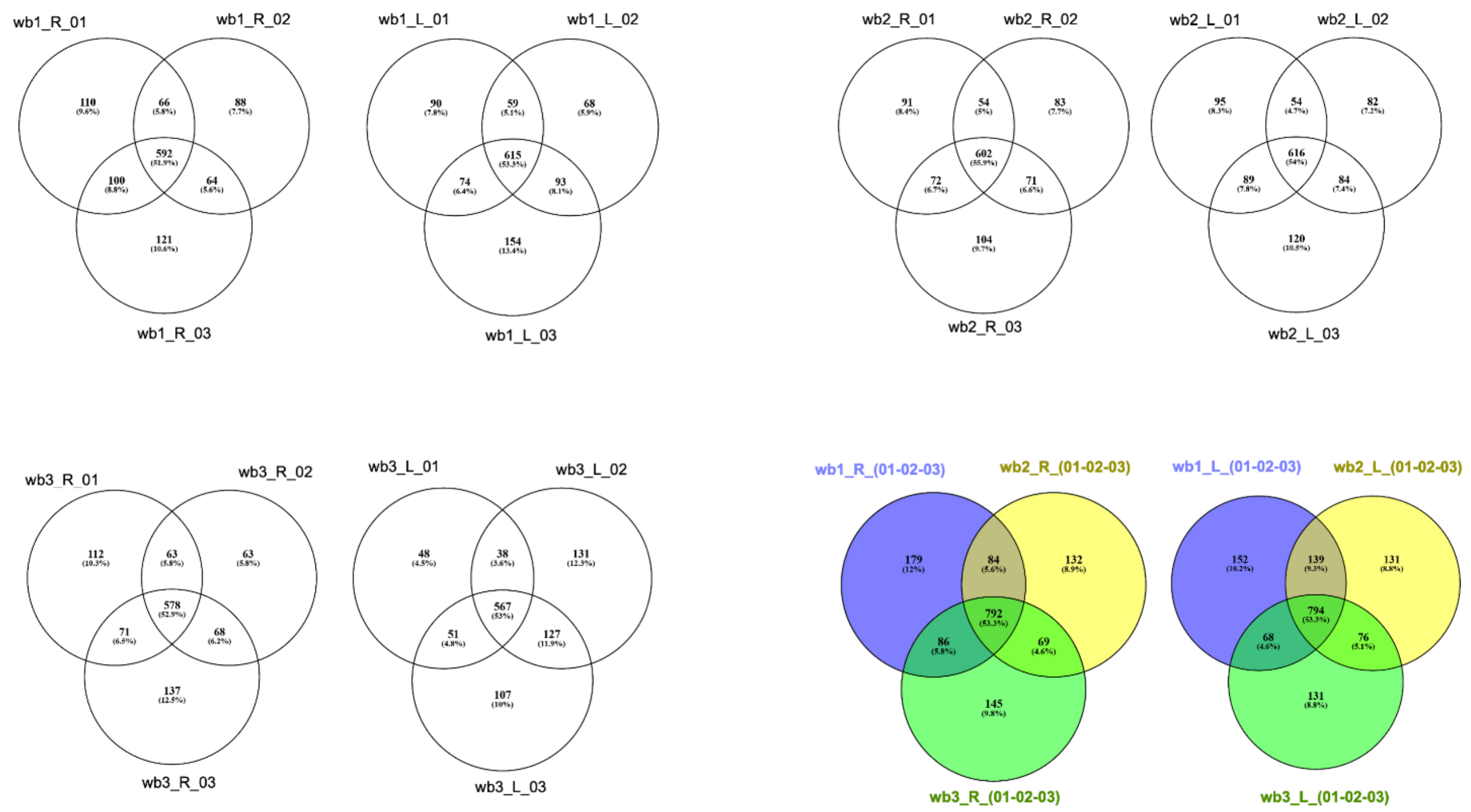


Figure 5. Venn diagrams showing overlap of protein identifications obtained in technical and biological replicates from wild boar (wb) samples. L, left; R, right.

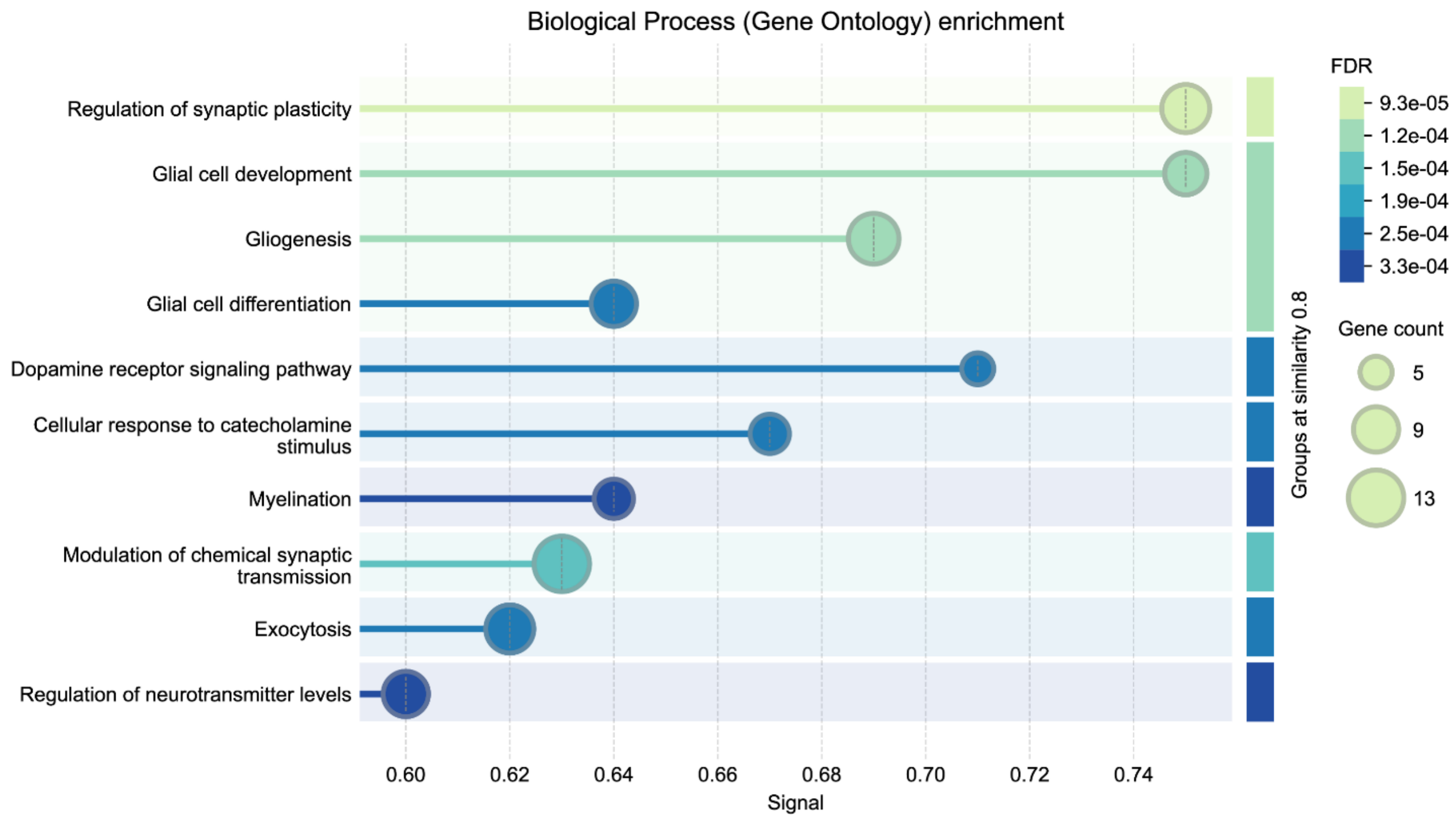


Figure 6. Biological processes enrichment differentially expressed proteins identified in the comparison of L + R M1 between wild boar and pig. Biological processes with significant FDR values and at least two counts are shown.

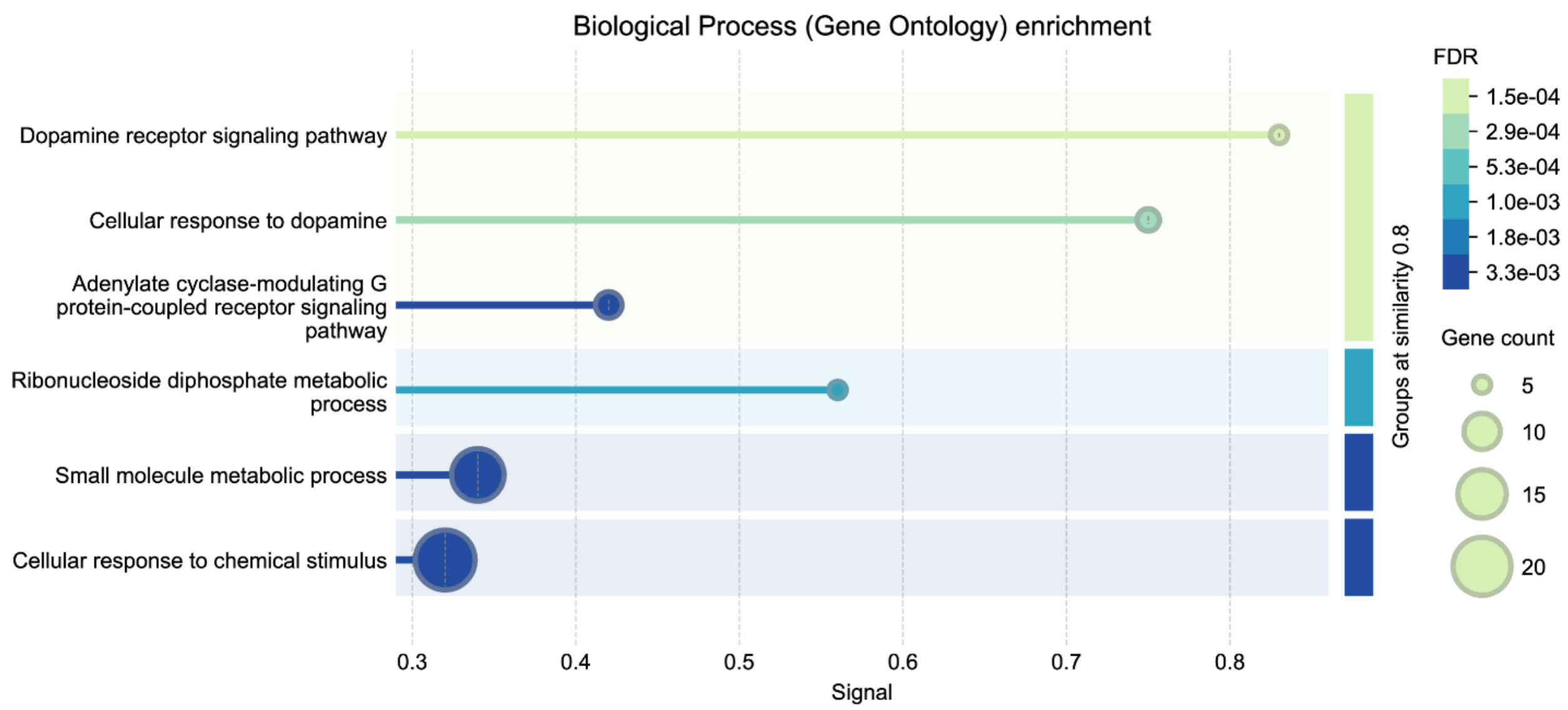


Figure 7. Biological process enrichment of only wild boar over-expressed proteins identified in the comparison of L + R M1 between wild boar and pig. Biological processes with significant FDR values and at least two counts are shown.

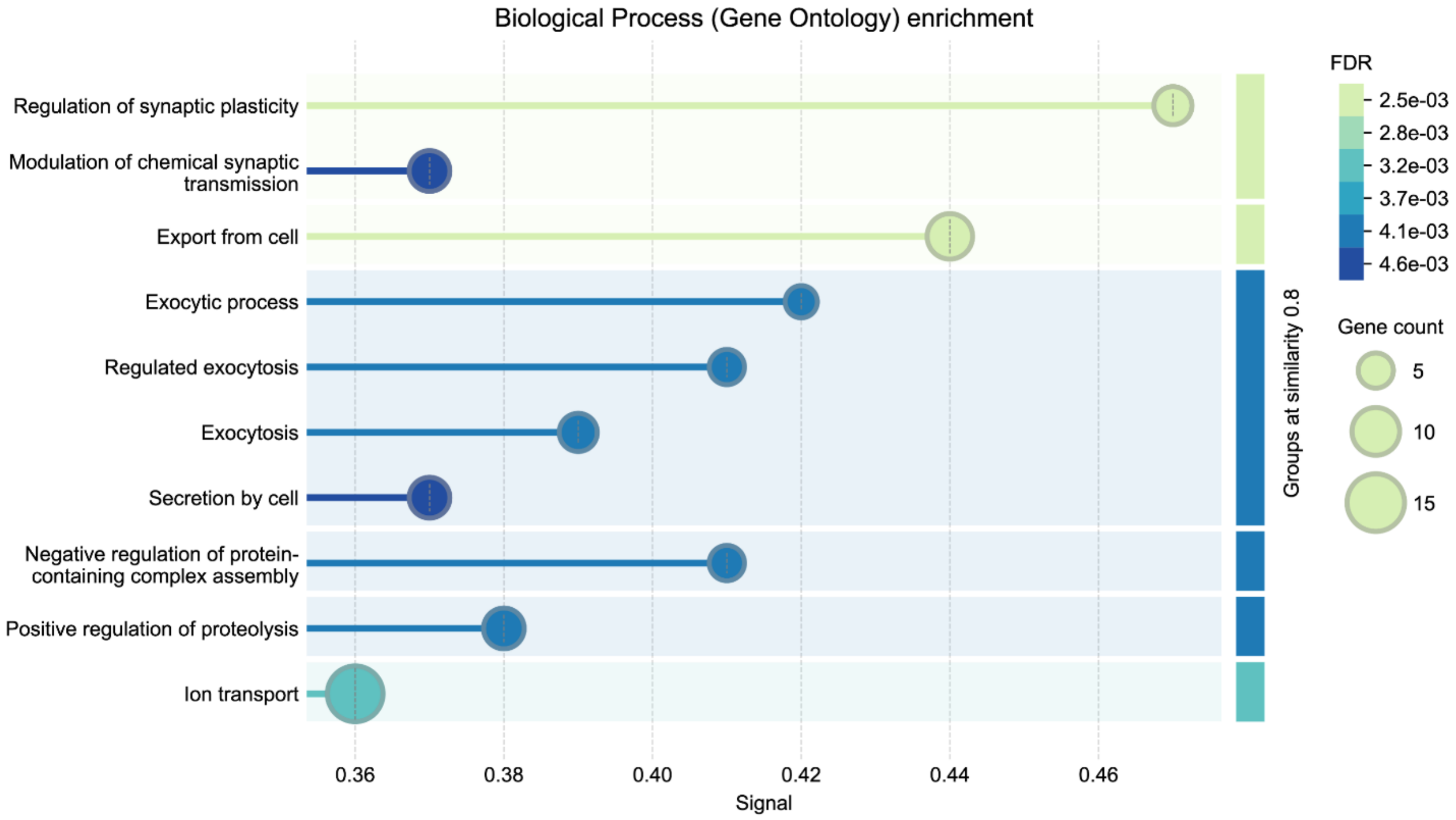


Figure 8. Biological processes enrichment of only pig over-expressed proteins obtained in comparison of L + R M1 between wild boar and pig. Biological processes with significant FDR values and almost two counts are shown.
