# Supplementary material for: Comparative cytoarchitectural and proteomic analysis of the primary motor cortex in pigs and wild boars reveals domestication associated changes
Source: PeerJ. 2026 Aug 18;14:e21640. doi: 10.7717/peerj.21640 (PMC13499532; doi:10.7717/peerj.21640)
Supplement: Supplementary file 2 [file peerj-14-21640-s002.docx]

| Group | Animal ID | Brain weight (g) |
| --- | --- | --- |
| Pigs | p1 | 113.8 |
|  | p2 | 110.7 |
|  | p3 | 106.5 |
|  | p4 | 112.0 |
|  | p5 | 106.4 |
|  | p6 | 111.0 |
| Wild boars | wb1 | 146.52 |
|  | wb2 | 141.61 |
|  | wb3 | 155.21 |
|  | wb4 | 157.73 |
|  | wb5 | 140.03 |
|  | wb6 | 151.88 |
