# Supplementary material for: Comparative cytoarchitectural and proteomic analysis of the primary motor cortex in pigs and wild boars reveals domestication associated changes
Source: PeerJ. 2026 Aug 18;14:e21640. doi: 10.7717/peerj.21640 (PMC13499532; doi:10.7717/peerj.21640)
Supplement: Supplementary file 3 [file peerj-14-21640-s003.docx]

Table 2. Antibodies

| **Antibody** | **Immunogen** | **Details** | **Dilution** |
| --- | --- | --- | --- |
| Anti-PV | Purified frog muscle parvalbumin. | Sigma-Aldrich, mouse monoclonal, Clone PARV-19, No. P3088, RRID: AB 477329 | 1:2000 |
| Biotinylated | Mouse IgG (H+L) | Vector Labs, Burlingame, horse,  Cat.n. BA-2000, RRID: AB 2313581 | 1:300 |
