# Supplementary material for: Comparative cytoarchitectural and proteomic analysis of the primary motor cortex in pigs and wild boars reveals domestication associated changes
Source: PeerJ. 2026 Aug 18;14:e21640. doi: 10.7717/peerj.21640 (PMC13499532; doi:10.7717/peerj.21640)
Supplement: Supplementary file 4 [file peerj-14-21640-s004.docx]

Table 3. The quantitative extracted morphometric parameters for each set of images acquired.

| Staining | Sections | Acquisition Scanner | Analysis Software | Morphometrics |
| --- | --- | --- | --- | --- |
| Nissl | 4 whole images *per* side *per* animal | Nano Zoomer Hamamatsu | Image Scope (Leica) | M1 Thickness (M1T, μm) |
|  | 4 whole images *per* side *per* animal | Nano Zoomer Hamamatsu | Stereo Investigator WSE (mbf, Bio.) | Density of cells (DC, cells/mm^2^) |
| Anti-PV | 4 whole images *per* side *per* animal | Nano Zoomer Hamamatsu | Image Scope (Leica) | Density of PV-immunoreactive (ir) neurons (DPVN, cells/mm^2^) |
